# Coexpression among eastern oyster host and microbiome genes suggests coordinated regulation of calcifying fluid chemistry

**DOI:** 10.1073/pnas.2521539123

**Published:** 2026-03-10

**Authors:** Andrea Unzueta-Martínez, Jennifer A. Delaney, Kate Morkeski, Abby Ross, Zhaohui Aleck Wang, Peter R. Girguis

**Affiliations:** ^a^Department of Organismic and Evolutionary Biology, Harvard University, Cambridge, MA 02138; ^b^Department of Marine Chemistry and Geochemistry, Woods Hole Oceanographic Institution, Woods Hole, MA 02543; ^c^Department of Ecology and Evolutionary Biology and Institute of Arctic and Alpine Research (INSTAAR), University of Colorado Boulder, Boulder, CO 80303

**Keywords:** shell, microbiome, oyster, host-microbiome

## Abstract

Marine animals that build shells, such as oysters, carefully regulate the chemistry of their internal calcifying fluids, but the molecular mechanisms behind this control, as well as whether microbes play a role in calcification, are poorly understood. To better understand oysters’ molecular mechanisms and the role of their calcifying-fluid microbes, we conducted experiments that simulated a tidal cycle, measured calcifying fluid pH and total dissolved inorganic carbon, and characterized host and microbial gene expression via transcriptomics. These experiments showed that calcifying fluid pH remained relatively stable throughout tidal pH fluctuations, with corresponding increases in oyster transcripts for ion transport and acid–base regulation. These data provide direct evidence that tidal fluctuations drive rapid changes in oyster calcifying fluid chemistry. Most surprisingly, increases in microbial transcripts related to nitrogen and sulfur cycling correlated to higher calcifying fluid DIC, and coexpression network analysis revealed patterns of gene expression that linked oyster immune and neural pathways to microbial redox processes, providing molecular evidence of potential host modulation of microbial metabolism. Together, these results reveal that oysters actively regulate their calcifying fluid pH over short timescales, and the endemic microbiome metabolic responses can yield metabolites that influence calcifying fluid pH, alkalinity, and ultimately calcification. These data offer a perspective on oyster physiological capacity and, most importantly, the potential role of microbes in oyster calcification. In light of ongoing changes in ocean pH and temperature, oysters provide a model for studying animal–microbial responses to environmental acidification and how their interactions may shape biomineralization.

## Significance

Oysters and many marine animals build shells by controlling the chemistry of extracellular fluids where minerals form, yet whether microbes in these fluids influence calcification remains unclear. We show that oysters maintain favorable conditions for mineral formation by regulating the carbonate chemistry of the shell-forming fluid, and that resident microbes respond to these changes by expressing nitrogen- and sulfur-cycling genes capable of altering pH, alkalinity, and carbonate availability. Many of these microbial transcripts were tightly correlated with oyster immune and signaling genes, suggesting that host and microbiome processes may be linked within the calcifying environment. These findings point to a host–microbiome interaction in the regulation of calcifying-fluid chemistry that directly links microbial activity to the carbonate chemistry underlying biomineralization.

Shallow and nearshore environments, such as estuaries, intertidal zones, and coastal ponds, are among the most physiologically demanding aquatic habitats, where environmental conditions, including pH, can fluctuate dramatically over short timeframes and small spatial scales. pH fluctuations in nearshore environments are primarily driven by variation of the carbonate system, which is controlled by a myriad of factors ranging from biological productivity (1) and terrestrial-riverine inputs (2, 3), to shelf processes [e.g., upwelling (4)]; and long-term climate signals (e.g., warming and ocean acidification (5). While numerous studies have examined the effects of projected future pCO_2_/pH scenarios (6), natural variability in nearshore environments can exceed long-term acidification trends, often exposing marine calcifiers to pH conditions well beyond those predicted for the coming century (7). Understanding how marine organisms regulate their internal pH and sustain biomineralization under such dynamic conditions sheds light on their physiological adaptations and will help predict future resilience.

To enable shell formation across a range of aquatic conditions, marine calcifiers such as oysters regulate the composition of an extracellular fluid known as the calcifying fluid. This fluid, where shell constituents such as calcium are stored (8), requires pH regulation to support the deposition of the calcium carbonate shell. In addition to facilitating shell formation, bivalves utilize their calcifying fluid and calcareous shells as buffers to mitigate the effects of acidic metabolic products such as CO_2_ and proton-equivalent byproducts that accumulate in extracellular fluids (9). Given the role of calcifying fluid in both biomineralization and acid–base homeostasis, understanding how oysters regulate pH within this compartment is essential for unraveling the physiological mechanisms underlying shell formation and acid–base balance in fluctuating environments.

The potential role of microorganisms in regulating calcifying fluid chemistry and contributing to calcification has been explored in some marine invertebrates, though there is paucity of mechanistic and experimental studies. In sponges, calcifying bacteria have been found at the site of spicule formation (10). In corals, their photosynthetic microbial partners have been implicated in the relationship between sunlight and calcification rates (11, 12). More recent work suggests that microbial consortia within the coral holobiont might engage in extensive metabolite exchange that may collectively regulate the chemical environment of calcification (13). In mollusks, previous work has described mucus-associated bacteria mediating calcium carbonate nucleation on the pedal mucus of Vermetid snails (14) and on the outer shell surfaces of cemented bivalves (15). Oyster shells exhibit unusual chalky and vesicular microstructures, prompting hypotheses that microbial activity, particularly by sulfate-reducing bacteria, may influence carbonate chemistry within the shell-forming environment (16–18). Subsequent studies have documented diverse microbial communities in the calcifying fluid and explored potential links between these microbes and shell mineralogy (19–21), these efforts largely relied on community composition or seawater manipulations. Our prior work identified microbial metabolic pathways in oyster calcifying fluids (22), including sulfate reduction and denitrification, that are known to alter pH, alkalinity, and inorganic carbon chemistry in microbial biofilms (23, 24), suggesting that calcifying-fluid microbes may have the functional potential to influence shell formation. However, the degree to which microbial metabolic activity influences carbonate chemistry in calcifying fluid and whether it integrates with host physiology to regulate calcifying-fluid chemistry remains unresolved. Across marine invertebrates, accumulating evidence links microbial activity to the regulation of mineral-forming environments, yet the functional roles and metabolic contributions of calcifying-fluid microbes remain unresolved.

In light of the physiological potential for microorganisms to influence calcifying fluid chemistry, here we investigate how calcifying-fluid-hosted microorganisms might influence the calcifying fluid environment to facilitate pH regulation and calcification. To this end, we used the eastern oyster (*Crassostrea virginica*) as a model system. Oysters are ideal for studying biomineralization due to their external calcified structures, which have been extensively characterized in terms of mineral composition, structure, and mechanical properties (25–28). A fully annotated genome and transcriptome are also available (29). Oysters have been found to host tissue-specific microbial communities across broad geographic ranges (30), including taxonomically rich assemblages in the calcifying fluid (22, 31) with genes linked to carbonate chemistry-altering metabolisms (22). To investigate how host and microbial processes in calcifying fluid respond to tidal seawater pH fluctuations, we quantified changes in calcifying-fluid chemistry alongside host and microbial gene expression. This integrative framework enabled us to evaluate whether microbial metabolic activity correlates with, and potentially contributes to, oyster-driven regulation of calcifying fluid chemistry.

## Results

**Fig. 1.**
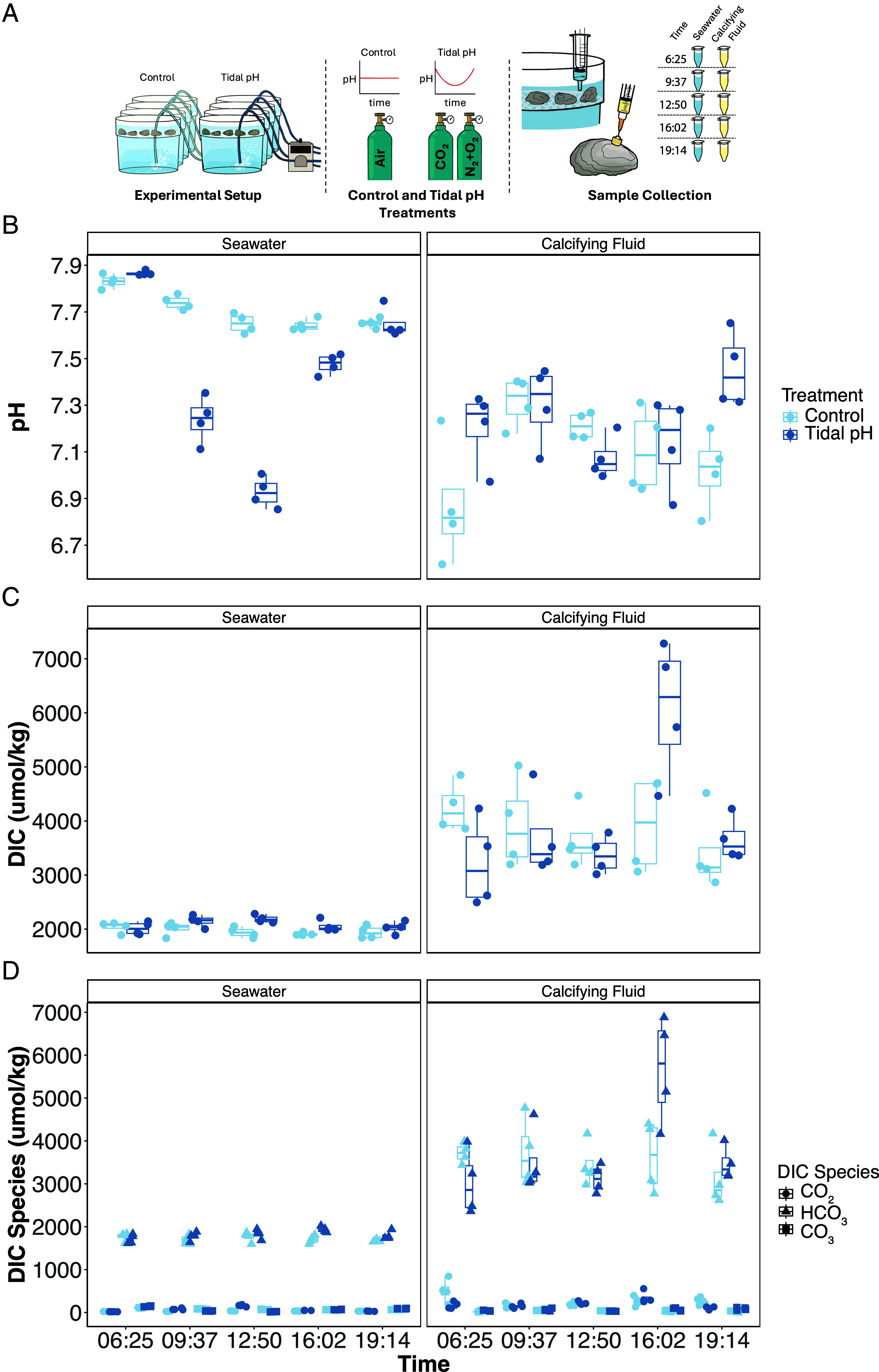
Overview of seawater and calcifying fluid chemistry in the control and tidal pH treatment levels. (*A*) Experimental design schematic showing independent aquaria maintained at either a constant Control pH or a diel-fluctuating Tidal pH regime. Control aquaria were bubbled with ambient air only, whereas tidal treatment aquaria were bubbled with CO_2_ and a certified N_2_/O_2_ gas mixture to achieve target seawater pH profiles. Oyster calcifying fluid and seawater were sampled at five time points (06:25, 09:37, 12:50, 16:02, 19:14) to measure (*B*) pH, (*C*) total dissolved inorganic carbon (DIC), and (*D*) DIC species (CO_2_(aq), HCO_3_^−^, and CO_3_^2−^). Significance was tested using two-sided *t* test and Benjamini–Hochberg used to correct for multiple comparisons. Assumptions of normality and equal variances were met for all time points (*P* > 0.05).

Twenty oysters were exposed to a tidal-like oscillation in seawater pH, and samples were collected at five time points over a 12-h cycle. The experiment consisted of eight aerated tanks. Four tanks were subject to a cyclic pH treatment (hereafter called “tidal pH,” in which we simulate the pH changes in seawater associated with daily tidal cycles) and four to constant pH. Each tank functioned independently, with one oyster and one seawater sample collected from each tank at every time point (n = 4). Seawater pH in the tidal treatment was manipulated by bubbling mixtures of CO_2_, N_2_, and O_2_ to reproduce the diel pH fluctuations typically observed in intertidal and subtidal habitats, where pH changes are driven by biological respiration and photosynthesis (11). Control tanks were bubbled with air to maintain oxygen concentrations and constant pH. This approach allowed us to simulate the carbonate chemistry dynamics of tidal cycles while keeping oysters continuously submerged and respiring. At each time point, we measured carbonate chemistry in both seawater and calcifying fluid and collected calcifying fluid via a sampling port (*Methods*) for host and microbial RNA sequencing. An additional twenty oysters were held at constant pH as controls (Fig. 1*A* and *SI Appendix*, Fig. S1).

### Decoupling of pH and DIC in Oyster Calcifying Fluid Over the Tidal Cycle.

**Fig. 2.**
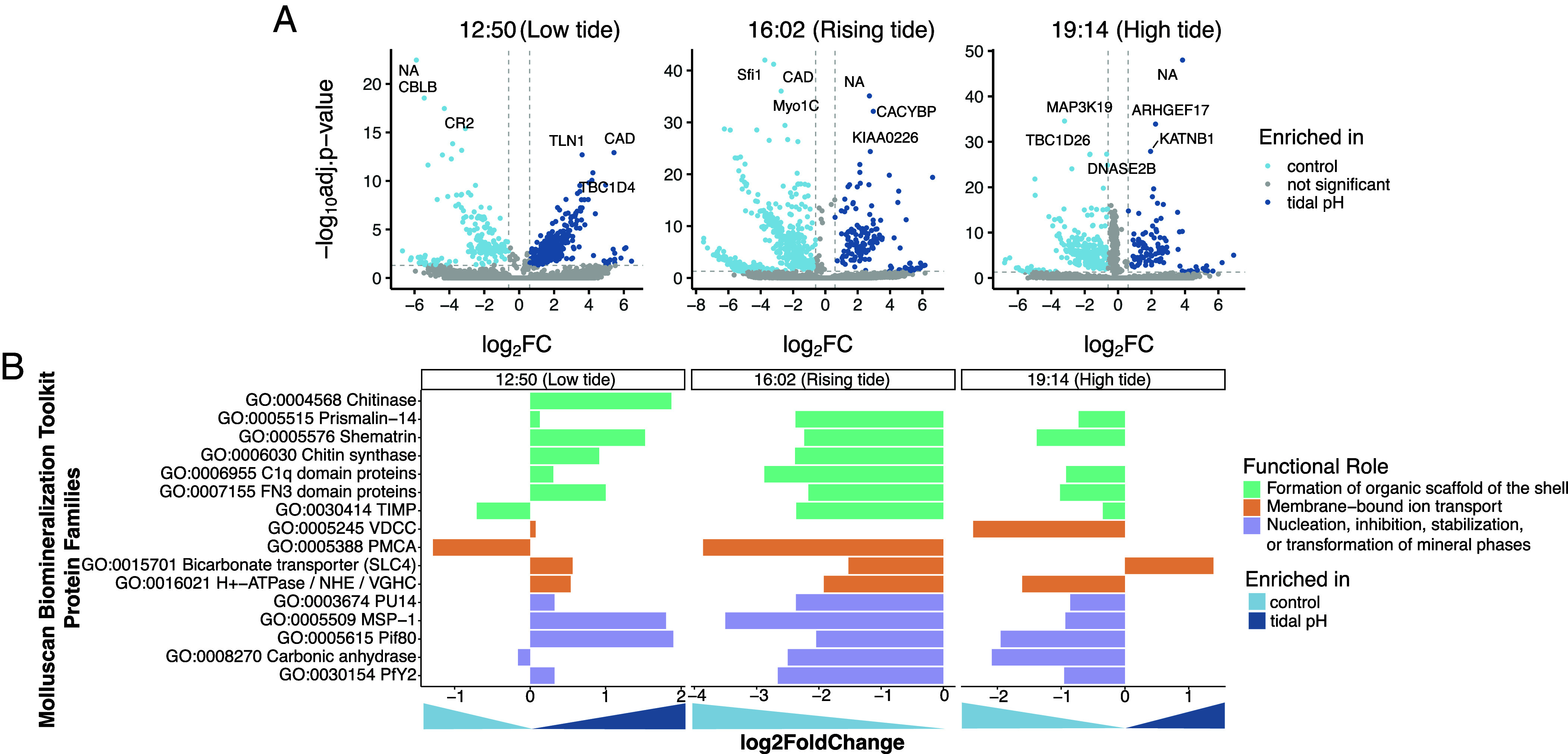
Differential expression analyses of oyster transcripts between control and tidal pH conditions. (*A*) Volcano plots comparing the expression of 55,005 oyster transcripts between conditions (log2FC) during the last three time points. Significance was assessed with the negative log of the adjusted *P* value < 0.05 using DESeq2. Colored dots highlight the transcripts significantly more abundant in the control condition (n = 403 at 12:50, n = 642 at 16:02, and n = 114 at 19:14, all in light blue) or tidal pH condition (n = 203 at 12:50, n = 149 at 16:02, n = 458 at 19:14, all in dark blue). (*B*) Differential expression of a curated set of biomineralization-associated genes grouped by functional role. Genes were classified as involved in membrane-bound ion transport, mineral phase regulation (e.g., nucleation, inhibition, or stabilization), or organic scaffold formation. Bars represent mean log2 fold change between treatments at each time point, with positive values indicating enrichment in tidal pH oysters.

Calcifying fluid pH (pH_CF_) remained relatively stable across the tidal cycle, with no significant differences between treatments among most time points (Fig. 1). For example, there was no significant difference between treatments at 12:50 (low tide, when external pH was lowest) or at 16:02 (rising tide, when calcifying fluid DIC peaked). Calcifying fluid pH was only significantly different in the tidal pH treatment at the 19:14 time point (high tide; *t* test, *P*_adj_ = 0.048, Fig. 2*B* and *SI Appendix*, Table S1). Within the tidal pH treatment, pH variation was minimal, with a modest difference between 12:50 and 19:14 (*P*_adj_ = 0.032, *SI Appendix*, Table S2).

Calcifying fluid DIC (DIC_CF_) concentrations were generally similar between treatments, except at the 16:02 time point when DIC_CF_ appeared elevated in the tidal pH group. Although this difference was not significant after multiple testing correction (*P*_adj_ = 0.15), DIC_CF_ varied significantly across time points within the tidal pH treatment (ANOVA, *P* = 0.0007), with 16:02 significantly higher than all other time points (*P*_adj_ < 0.005, Fig. 1*C* and *SI Appendix*, Table S4). The DIC_CF_ peak occurred just after the lowest seawater pH and was not accompanied by a shift in pH_CF_.

Across all samples, the majority of DIC_CF_ existed as bicarbonate ions (HCO_3_^−^), with lesser amounts of dissolved CO_2_ and carbonate ions (CO_3_^2−^), consistent across treatments and time points (Fig. 1*D* and *SI Appendix*, Tables S5 and S6).

### Oyster Transcripts Suggest Ion Pumps May Regulate Calcifying Fluid Chemistry.

To examine biological mechanisms underlying the DIC_CF_ spike at 16:02, we analyzed host and microbial transcriptomes from calcifying fluid at 12:50, 16:02, and 19:14. These time points span the window of chemical variability. Host gene expression showed distinct treatment effects across time points (Fig. 2). At 12:50 and 16:02, control oysters exhibited a larger set of significantly enriched transcripts, whereas at 19:14, more transcripts were enriched under tidal pH conditions.

We next examined a curated set of biomineralization genes associated with carbonate chemistry and pH regulation, categorized as ion transporters, matrix proteins, or crystal nucleation regulators (Fig. 2*B*), following the framework of Zhuoquing et al. (32). These genes were consistently downregulated in the tidal pH treatment at 16:02. PMCA and H^+^-ATPase, along with Shematrin and FN3 domain proteins, were more highly expressed in the control. Full transcriptome-wide differential expression and GO enrichment results support these trends, showing shifts in ion transport ATP hydrolysis, GTPase activity, and sodium:bicarbonate symporter activities (*SI Appendix*, Fig. S2).

### Microbiome Transcriptional Changes in Response to Calcifying Fluid Chemistry.

**Fig. 3.**
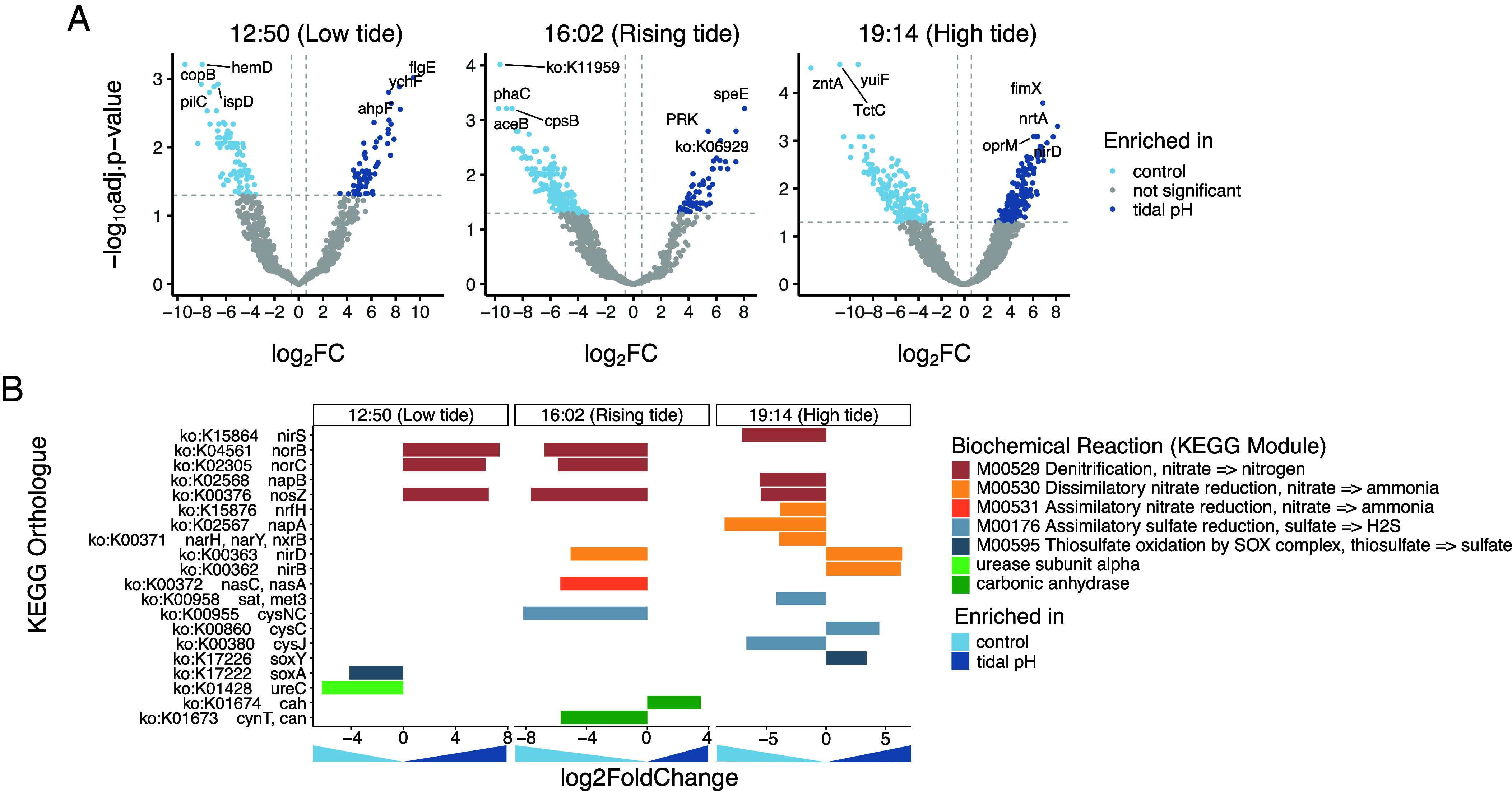
Differential expression analyses of microbiome transcripts between control and tidal pH conditions. (*A*) Volcano plots comparing the expression of 3,810 microbiome transcripts between conditions (log2FC) during the last three time points. Significance was assessed with the negative log of the adjusted *P* value < 0.05 using DESeq2. Colored dots highlight the transcripts significantly more abundant in the control condition (n = 87 at 12:50, n = 200 at 16:02, and n = 150 at 19:14, all in light blue) or tidal pH condition (n = 63 at 12:50, n = 62 at 16:02, n = 168 at 19:14, all in dark blue). (*B*) Differentially expressed transcripts of genes involved in biochemical reactions known to alter local carbonate chemistry. Colors represent the biochemical reaction each gene is involved in.

We analyzed changes in microbial gene expression in response to changes in tidal pH. Sequencing of procedural negative controls and mock communities confirmed data reliability (*SI Appendix*, Fig. S3). Microbial gene expression shifted across time points and treatments (Fig. 3). At 12:50 and 16:02, there was a greater number of significantly enriched transcripts in control oysters; while at 19:14, there was a greater number of significantly enriched transcripts in tidal pH oysters. Transcripts enriched in the tidal pH treatment included stress response genes (ahpF, oprM) and polyamine synthesis (speE), as well as nitrogen metabolism (nrtA, nirD) and carbon fixation (PRK). We also identified microbial genes linked to carbonate-modifying processes (Fig. 3*B*). Genes for denitrification, nitrate reduction, and sulfur reduction were upregulated in the tidal pH group, especially at 16:02 and 19:14. In contrast, genes related to carbonic anhydrase and sulfur oxidation were more abundant in controls. KEGG enrichment supported these trends, with nitrogen and sulfur metabolism enriched in tidal pH samples and fewer pathways enriched in controls (*SI Appendix*, Fig. S4).

Regression analyses showed no microbial gene expression associations with external seawater pH or DIC, nor with calcifying fluid pH. However, several transcripts (rnfA, nirA, sir) were significantly associated with calcifying fluid DIC (*SI Appendix*, Fig. S5), indicating microbial expression correlated strongly to internal chemistry.

### Coexpression Among Host and Microbiome Genes Suggests Coordinated Regulation of Calcifying Fluid Chemistry.

**Fig. 4.**
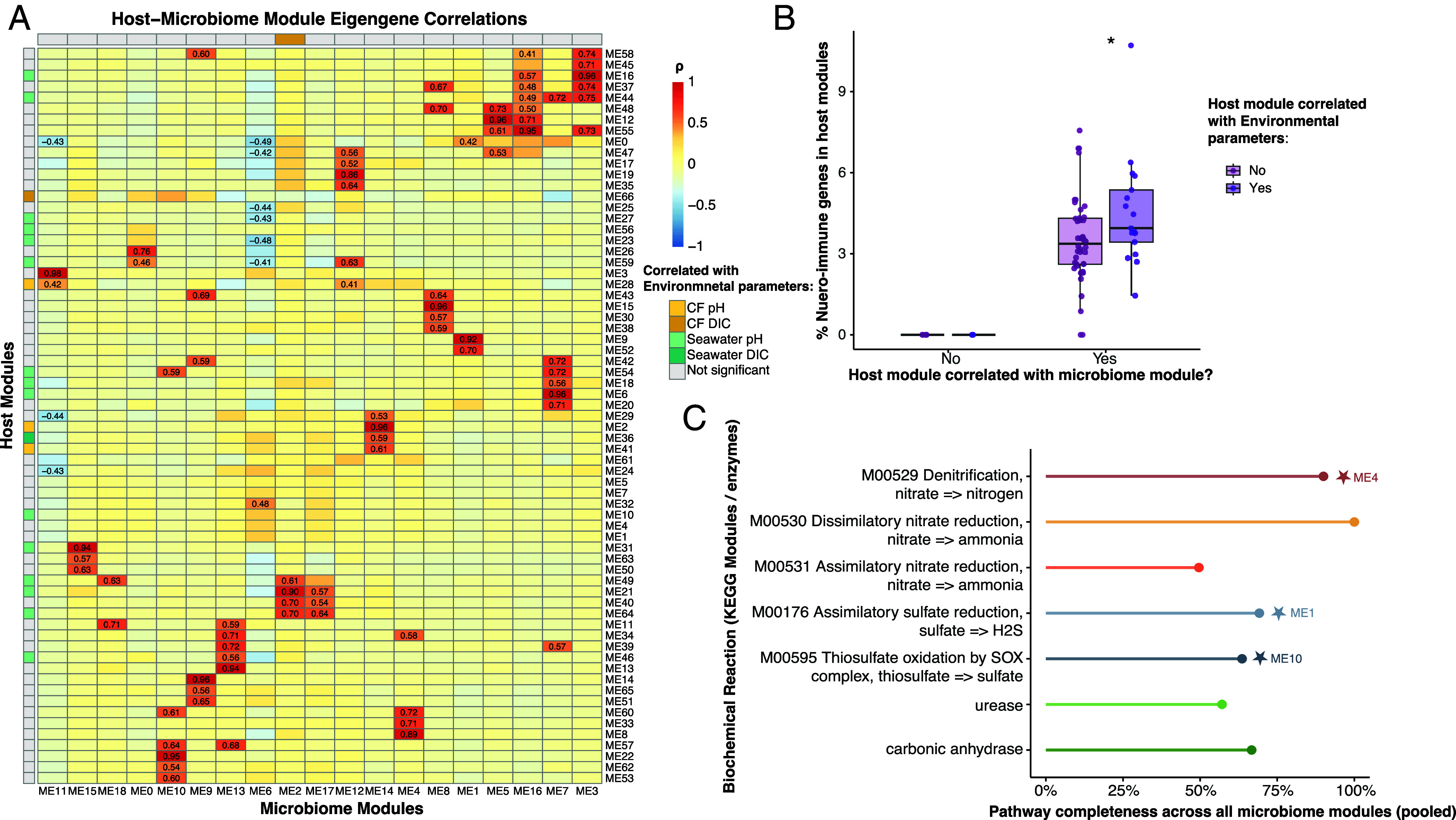
Host–microbiome coexpression and pathway relationships. (*A*) Heatmap showing module eigengene correlations between host and microbiome WGCNA modules. Each cell represents a Pearson correlation coefficient (ρ) between a host and microbiome module eigengene. Numerical values are shown for statistically significant correlations (*P* < 0.05). Modules correlated with environmental parameters (calcifying-fluid pH or DIC, seawater pH or DIC) are annotated along the axes. (*B*) Boxplot showing the proportion of host genes annotated with Gene Ontology (GO) terms related to host–microbe communication (e.g., neuro-immune or signaling processes) in modules that are (purple) or are not (gray) significantly correlated with at least one microbiome module. GO terms were classified as “host–microbe communication” if they fell under the parent categories “symbiosis, encompassing mutualism through parasitism” (GO:0044403), “multi-organism signaling” (GO:0051705), or “response to symbiont” (GO:0044416) within the Biological Process ontology (summarized in *SI Appendix*, Table S8). (*C*) Pathway completeness of key biochemical reactions and enzyme complexes known to alter local carbonate chemistry across all microbiome modules (pooled). Completeness values represent the percentage of KEGG orthologs (KOs) detected relative to the total number of KOs defining each pathway. For enzyme complexes such as urease and carbonic anhydrase, completeness was instead based on the detection of structural subunits (for urease) or enzyme types (for carbonic anhydrase) required for catalytic activity. Stars mark the microbiome modules (e.g., ME4, ME1, ME10) in which these pathways were significantly enriched. Detailed enrichment analysis results are provided in *SI Appendix*, Table S10.

To assess coordinated expression between the oyster and its microbiome, we conducted independent weighted gene coexpression network analyses (WGCNA) for the host and microbial transcriptomes, identifying 67 host and 19 microbiome modules of coexpressed genes (Fig. 4*A*). We first correlated each module eigengene to environmental parameters (seawater and calcifying-fluid pH and DIC; *SI Appendix*, Fig. S6), and we identified 19 host modules and one microbiome module that were significantly correlated to environmental parameters (*P* < 0.01; *SI Appendix*, Fig. S6). We then correlated host and microbiome modules with each other and found several significant module pairs that covaried independently of environmental parameters (*P* < 0.01; Fig. 4*A*), suggesting that shared gene expression patterns may reflect biological coordination rather than parallel responses to external pH conditions. Host modules correlated with microbiome modules contained genes annotated to neuro-immune processes, which were entirely absent from uncorrelated modules (Fig. 4*B* and *SI Appendix*, Table S8). Among these host modules, those that were also correlated with environmental variables contained a higher percentage of neuro-immune genes than those that were not (Wilcoxon rank-sum test, W = 475.5, *P* = 0.048; Fig. 4*B* and *SI Appendix*, Table S9). Microbiome modules collectively encoded multiple biochemical pathways capable of altering carbonate chemistry, including denitrification, nitrate and sulfate reduction, and thiosulfate oxidation, as well as enzymes urease and carbonic anhydrase (Fig. 4*C*). Several of these pathways were near complete across the pooled microbiome modules, indicating broad metabolic potential for nitrogen and sulfur transformations. Pathway enrichment analyses further identified specific microbiome modules significantly enriched in these KEGG processes (Fig. 4*C*, indicated by stars; *SI Appendix*, Table S10), including ME4 for denitrification, ME1 for assimilatory sulfate reduction, and ME10 for thiosulfate oxidation.

## Discussion

Our study revealed evidence of that microbes found in calcifying-fluid express metabolic genes known to influence local pH and alkalinity and that abundance of these transcripts positively correlated to oyster neuro-immunological pathways, suggesting potential physiological coordination between host and microbiome to shape calcifying fluid chemistry. There were four key findings: first, coexpression network analysis revealed modules that linked host immune genes (e.g., Toll-like receptor signaling) with microbial redox genes (e.g., sulfate and nitrate reduction), which as mentioned point to potential coordination between microbial recognition pathways in the host and microbial processes known to influence carbonate chemistry. Second, microbial gene expression patterns aligned more closely with calcifying fluid DIC than with external conditions and included transcripts for nitrogen and sulfur metabolic processes that are known to influence pH, alkalinity, and presumably calcifying fluid chemistry and shell calcification. Third, oyster genes associated with carbonate chemistry and pH regulation were highly expressed at low tide and downregulated at the time of peak internal DIC, suggesting an early activation of carbonate-handling pathways during acid stress, followed by a tapering of transcriptional investment as internal buffering needs subside. Fourth, calcifying fluid pH remained stable while DIC spiked transiently during the rising tide, suggesting active internal regulation rather than passive tracking of seawater chemistry. Together, these findings highlight a dynamic interplay between host physiology and microbial function during pH fluctuations experienced during the tidal cycle.

It should be noted this experimental design ensured that oysters remained submerged in oxygen-replete water, minimizing the possibility of prolonged shell closure that could result in marked changes in calcifying fluid chemistry and contribute to correlated expression patterns between the host and microbiome. Moreover, efforts were also made to minimize any perturbations such as vibrations that would influence oyster behavior. Though tracking internal oxygen dynamics was beyond the scope of the present study, the strength and specificity of the observed correlations over all the tested individuals and conditions suggest that individual behaviors were unlikely to have influenced the observed trends.

### WGCNA Reveals Correlated Host–Microbiome Responses to Tidal pH Fluctuations.

Network analysis revealed transcriptional correlation between the oyster host and its microbiome that would not be apparent from differential expression analysis alone. Independent WGCNA analyses of the host and microbiome identified 67 host and 19 microbial modules of coexpressed genes. Correlations among these modules revealed multiple significant host–microbiome module pairs that covaried independently of environmental parameters (Fig. 4*A*), suggesting that shared gene expression patterns could arise from biological coordination rather than simple parallel responses to external pH or DIC changes. These patterns of correlated expression echoe findings from other holobiont systems where host and microbial gene expression are tightly linked. In *Trichodesmium* filamentous cyanobacterial colonies, for example, there is synchronized diel gene expression between the cyanobacterial host and its microbial partners, with coregulation of pathways involved in nitrogen, carbon, and vitamin B_12_ cycling (33). Similarly, gene expression in aphid bacteriocytes and their *Buchnera* symbionts is highly interdependent, reflecting a dynamic metabolic “tug-of-war” shaped by host genotype and symbiont density (34).

Host modules that were significantly correlated with microbiome modules contained a higher proportion of genes involved in neuro-immune functions, such as Toll-like receptor signaling, antimicrobial peptide regulation, and calcium-dependent neuronal signaling, compared to host modules that were not correlated with microbiome modules (Fig. 4*B* and *SI Appendix*, Table S8). Moreover, these genes represented a higher proportion of transcripts in host modules that also correlated with environmental parameters (Fig. 4*B* and *SI Appendix*, Table S9), suggesting that host–microbiome coordination may be more important during environmental perturbations. This pattern suggests that communication between the immune and nervous systems may help coordinate host–microbe interactions within the calcifying fluid. Previous studies have highlighted similar links between immunity and biomineralization in oysters (though they did not examine the presence or activity of microbes). Ivanina et al. (35) showed that hemocytes mediate both immune defense and shell formation, with species-specific trade-offs in cellular investment. Likewise, Johnstone et al. (36, 37) demonstrated that hemocytes and mantle epithelial cells cooperate to deposit shell material and secrete extracellular-matrix proteins during mineralization. Given the role that hemocytes play in immunity, these data provide circumstantial support for the plausible involvement of microbes in shell deposition and/or dissolution.

The presence of neural signaling genes that are often linked to host–microbe communication within these same host modules further raises the possibility that neural regulation may also play a role in modulating host–microbe interactions within the calcifying fluid (Fig. 4*B* and *SI Appendix*, Table S8). This notion is consistent with emerging evidence from other invertebrate systems showing that neural signals can influence immune and epithelial function at host–microbiome interfaces (38). Boettiger et al. (39) proposed that neurosecretory pathways in gastropods contribute to shell patterning and structural organization, linking nervous system activity to biomineralization and shell repair processes. While the role of neural control in calcifying-fluid regulation remains largely unexplored, the co-occurrence of neural, immune, and microbial transcripts in correlated modules suggests a potentially integrated internal signaling network that supports homeostasis under fluctuating environmental conditions.

All microbiome modules showed significant correlations with host modules (Fig. 4*A*). Across these modules, microbial genes displayed high pathway completeness for metabolic processes capable of altering carbonate chemistry, and several modules were specifically enriched for nitrogen- and sulfur-cycle pathways such as denitrification, nitrate reduction, and thiosulfate oxidation (Fig. 4*C*). Together, these patterns suggest that the oyster and its associated microbes may mount coordinated metabolic responses to environmental change within the calcifying fluid. Prior work aimed to link sulfate-reducing bacteria to the mineralized chalky deposits found in oyster shells either by experimentally manipulating sulfate reducers in surrounding seawater (20) or by comparing 16S profiles of calcifying fluid microbiomes of oysters with and without chalk formation (21). Although these studies did not find a direct link between sulfate-reducing bacteria and chalk formation, they highlight the possibility that microbial processes may influence carbonate chemistry within the shell-forming environment more broadly. Our gene-expression analyses address this gap by providing molecular evidence that calcifying-fluid microbes have the functional capacity to influence carbonate chemistry in correlation with host gene expression.

The correlation of host neuro-immune transcripts with microbial genes involved in biogeochemical cycling relevant to biomineralization suggests that host and microbial processes may respond jointly within the calcifying fluid. Future work will be needed to disentangle the individual and synergistic contributions of the nervous system, immune pathways, and microbiome to the regulation of calcifying fluid chemistry and the process of biomineralization. These network patterns complement the independent microbial, host, and chemical analyses described in the subsequent sections.

### Microbial Metabolism Responds to Host-Modulated Internal Chemistry.

Our transcriptomic data reveal functional plasticity at the gene expression level and demonstrate that microbial communities within the oyster calcifying fluid exhibit dynamic and functionally relevant transcriptional responses to the tidal pH treatment. Though previous work has shown that microbiome composition (via 16S sequencing) on marine bivalves can shift compositionally in response to low seawater pH (40, 41), our data provide insights into the functional changes that take place over the simulated tidal cycle. Genes enriched in the tidal pH treatment (Fig. 3*A*), including ahpF, oprM, and speE, are linked to oxidative stress defense and pH buffering, indicating microbial sensitivity to acid–base changes within the calcifying space. This mirrors host responses to acidified seawater, which induce acidosis in oyster hemolymph (42), and suggests that microbes and host tissues may face similar internal stress. The enrichment of PRK, a gene involved in carbon fixation, points to shifts in microbial energy metabolism under variable pH, a response pattern also seen in sediment (43) and bovine rumen (44) microbial communities under environmental stress.

Several microbial genes involved pathways that influence the carbonate system, including denitrification and sulfate reduction, were differentially expressed between treatments (Fig. 3*B*), suggesting that microbial metabolism may contribute to local pH buffering in the calcifying fluid (16, 17, 22). Though the capacity of host-associated microbes to alter internal carbonate chemistry remains unresolved, microbial biofilms are known to influence local geochemistry and facilitate carbonate precipitation in systems like stromatolites (23). We also identified transcripts for carbonic anhydrase (EC 4.2.1.1) and urease (EC 3.5.1.5) (Fig. 3*B*), enzymes that enhance carbonate availability in microbial cultures (45, 46). Their expression in the calcifying fluid suggests that microbes may support host pH and DIC regulation.

Finally, microbial gene expression correlated with calcifying fluid DIC, but not with external seawater chemistry (*SI Appendix*, Fig. S5), underscoring microbial sensitivity to host-modulated conditions. This parallels previous findings in oysters, where microbiome composition tracked host physiology more closely than seawater pH (41), supporting the view that the microbiome contributes to an integrated system of carbonate chemistry regulation.

### Host Transcriptomes Reveal Staged Physiological Responses to Tidal pH Variability.

The oyster’s transcriptomic response to tidal pH fluctuations suggests a staged regulatory strategy for carbonate homeostasis. Ion transport and acid–base regulation genes were upregulated at 12:50 (simulated low tide), when seawater pH was lowest, and declined by 16:02 and 19:14 (Fig. 2*B*). This pattern reflects energy-efficient regulation, in which costly transcriptional responses are activated only when needed (42, 47). The DIC spike at 16:02 likely reflects a host-driven compensatory response, potentially involving transcriptional activation and bicarbonate mobilization from shell dissolution to buffer internal pH, similar to hypoxia responses where closed-valve oysters use shell carbonate to counteract acidosis (9, 42, 48). Such buffering may begin rapidly and nontranscriptionally, while ion transport supports sustained recovery.

The continued enrichment of ion transport and regulatory functions at high tide (19:14) suggests that these compensatory processes are not transient but may persist to reestablish or maintain homeostasis following exposure to low pH. The expanding set of enriched functions over time reflects the oyster’s transcriptional plasticity in adjusting to fluctuating conditions. This aligns with findings that mollusks reallocate energy and adjust immune activity under environmental stress (49, 50), and underscores the capacity of oysters to regulate their internal environment through fine-tuned, time-sensitive gene expression.

### Calcifying Fluid pH and DIC Decoupled Over the Tidal Cycle.

Our results demonstrate a clear decoupling between pH and DIC in the oyster’s calcifying fluid during a simulated tidal pH cycle. While pH remained relatively stable, DIC spiked at 16:02, shortly after exposure to the lowest seawater pH (Fig. 1). Similar decoupling patterns have been observed in corals: In *Porites* DIC_cf_ peaked in summer while pH_cf_ was highest in winter (51), and in *Pocillopora damicornis*, DIC_cf_ increased as pH_cf_ declined under acidified conditions (47). These examples suggest that independent regulation of DIC and pH supports stable acid–base balance in CF. Our high-resolution dataset captures a comparable pattern unfolding over 12 h and reveals a short delay between external acidification and internal DIC response, temporal dynamics unresolved by longer studies. Although bivalve work has linked tidal cycles to shell deposition timing via growth increments (52, 53), this is the first direct evidence that simulated tidal fluctuations also drive rapid changes in calcifying fluid chemistry. Our findings confirm oysters among marine calcifiers that actively regulate carbonate chemistry on short timescales.

## Conclusion

This study reveals that oyster calcifying fluid harbors active microbial communities that potentially influence factors that relate to biomineralization. Furthermore, coexpression analysis identified modules linking host immune and neural signaling with microbial metabolism, pointing to a potentially synergistic role of both host and microbe in shaping acid–base regulation. Microbial gene expression, including metabolic processes that influence carbonate chemistry, tracked more closely with internal DIC_CF_ than with external conditions. Host gene expression exhibited a staged transcriptional response, with carbonate-handling pathways activated during low tide and downregulated as internal buffering needs subsided. Despite external acidification, pH_CF_ remained relatively stable while DIC_CF_ spiked, suggesting host-driven buffering through bicarbonate mobilization and ion transport. Together, these findings highlight host–microbiome interactions, including the potential for coordination that supports carbonate homeostasis, and lay the groundwork for future studies on neuro-immune-microbial contributions to biomineralization.

## Methods

### Oyster Collection and Luer-Lock Port Installation.

Adult, diploid, *C. virginica* (88- to 100-mm shell length), were purchased from Thatch Island Oysters (41°42′37.6″N 70°18′18.5″W) in Barnstable, Massachusetts (*n* = 40). The oysters were transported on ice to Harvard University, where they were allowed to recover for 24 h in a rearing tank with filtered seawater and fed 20 mL of a 1% Shellfish Diet 1800 solution, following best practices outlined in Helm and Bourne, 2004. The seawater used in our rearing tank and experimental tanks was collected from Hampton State Pier in Hampton, New Hampshire, and had a salinity of 34 PSU (practical salinity units). Seawater was UV-sterilized, filtered through a 30-µm filter, chilled to 6 °C, and stored in a recirculating 7,500-L polyurethane tank. The seawater was warmed to 18 °C before putting the oyster in the rearing tank.

Luer-lock ports were installed in the right valve of each oyster to access their calcifying fluids, similar to methods previously used (8, 41, 54, 55), but with modifications to optimize the drilling precision and sterility necessary for microbiological investigations. A high-precision DREMEL tool (model 8220; Dremel, Racine, WI) with a model 225 flexible shaft extension was used to drill a 2-mm diameter hole on the left valve of each oyster, approximately 2 cm from the hinge. Before drilling, 70% ethanol was used to sterilize the tool bits and shell surface, as well as to remove excess calcium carbonate powder from the drill site. To prevent damage to the mantle tissue, we visually inspected it through the drilled hole. After confirming that the mantle remained intact, ethanol-sterilized luer-lock ports were installed. We secured the ports to the oyster shell using waterproof Starbond® glue, commonly used in aquascaping and coral propagation. The oysters were placed in containers with enough seawater to cover their open valves while keeping the drilled area dry in order to allow the glue to fully set. This setup ensured proper adhesive curing while minimizing stress to the oysters. After 24 h, we returned the oysters to their rearing tanks and allowed them to recover for 2 wk before conducting any experiments.

### Tidal pH Exposure Experiment.

To simulate tidal fluctuations in seawater pH, we used mass flow controllers (Alicat Scientific, Inc., Tucson, AZ) to bubble CO_2_ at a rate of 1 mL/min to lower seawater pH for 5 h and 35 min, followed by an O_2_/N_2_ gas mixture (21% Oxygen, 79% Nitrogen) at 175 mL/min for another 5 h and 35 min to restore pH levels over a full tidal cycle (12 h and 50 min). Control conditions were maintained by bubbling room air into the aquaria. We used ¼” outer diameter nylon tubing and 1.5″ by 3″ air stones with ¼″ NPT PE fitting (model: ALS5, Pentair Aquatic Eco-Systems, FL) for gas delivery into the aquaria. The experiment was conducted in 20 L polycarbonate aquaria (Choice, WebstaurantStore, United States), with four replicate tanks per treatment and five oysters per tank to ensure four independent replicates per time point per treatment (Fig. 1*A* and *SI Appendix*, Fig. S1). Each aquarium was maintained as an independent closed system, with seawater from the same initial batch distributed separately among tanks to ensure replication without shared water. Every 3 h and 12 min, seawater and calcifying fluid were sampled for pH, conductivity, and dissolved inorganic carbon (DIC). Additionally, we collected calcifying fluid for RNA transcriptomic analyses from the final three time points. Sampling involved multiple researchers working in concert to rapidly collect seawater and calcifying-fluid samples simultaneously to ensure that all oysters were sampled within minutes of each target time point.

### Seawater Chemistry.

At each time point, seawater pH was measured with an InLab Expert Pro-ISM pH electrode (Mettler-Toledo GmbH, Schwerzenbach, Switzerland) calibrated with pH 7.01 and pH 10.01 NBS buffers (for calibration slope) and Dickson seawater Certified Reference Material (for calibration intercept). Seawater conductivity was measured with an InLab 731-ISM conductivity probe (Mettler-Toledo GmbH, Schwerzenbach, Switzerland). Seawater was collected in 250 mL borosilicate serum bottles, poisoned with 500 μL saturated ZnCl_2_, sealed with air-tight aluminum-crimped butyl rubber stoppers, and refrigerated at 4 °C until DIC analysis.

### Calcifying Fluid Chemistry.

At each time point, we used sterile 5-mL syringes and flexible plastic tubing oral gavage needles (20-gauge, 38 mm length, 1.75 mm tip diameter) to collect approximately 1.5 mL of calcifying fluid per individual. An aliquot of 500 μL was decanted in a 1.5 mL reaction tube to measure the electrical conductivity (μS/cm) using a MI-915 Micro-Conductivity Electrode and the pH using a MI-406 Flat Membrane pH Electrode (both from Microelectrodes Inc.®, Bedford, NH). The temperature (°C) was also measured using a temperature probe ATC NTC 30 k Ohm (Mettler Toledo, Billerica, MA). All three probes were connected to a SevenExcellence pH and Conductivity Meter S470 (Mettler Toledo, Billerica, MA). Another 500 μL of calcifying fluid was diluted in 2.5 mL of RNAse-free water, then filter sterilized through a 0.22-μm syringe filter (33 mm diameter, PES material, 2 μm pore size, EZFlow®, NH) while being transferred to an air-tight glass syringe. Syringe plungers were greased with Apiezon L grease (M&I Materials Ltd., Manchester, United Kingdom). Glass syringes were refrigerated at 4 °C until DIC analysis.

### Dissolved Inorganic Carbon Measurements.

Seawater and calcifying fluid DIC were measured using an Apollo SciTech DIC auto-analyzer at Woods Hole Oceanographic Institution, and measurements were calibrated using Certified Reference Materials (CRM) provided by Dr. A. Dickson at Scripps Institute of Oceanography. The seawater carbonate chemistry calculations were made with the Seacarb R package, analogous to CO2SYS software (56), using constants K1 and K2 by Roy et al. (57), the KHSO_4_ dissociation constant from Dickson (58), and the borate relationship from Lee et al. (59).

### Calcifying Fluid Chemistry Statistical Analyses.

To compare calcifying fluid pH and DIC between control and tidal pH treatments at each time point, we used two-sample unpaired *t* tests. At each time point, one oyster was sampled from each of four replicate tanks per treatment (n = 4 per treatment). Each tank was treated as an independent experimental unit. We verified normality using the Shapiro–Wilk test and homogeneity of variance using Levene’s test prior to conducting each *t* test. To account for multiple comparisons across time points, *P*-values were adjusted using the Benjamini–Hochberg false discovery rate (FDR) correction. To determine whether pH and DIC in the tidal pH treatment varied significantly across the tidal cycle, we used a one-way ANOVA with time point as a fixed factor. Assumptions of normality and homogeneity of variance were tested using the Shapiro–Wilk and Levene’s tests, respectively. Pairwise differences between time points were assessed using Tukey’s HSD post hoc tests.

### Calcifying Fluid RNA Extraction and Sequencing.

For each oyster sampled at the last three time points, we dispensed 500 μl of calcifying fluid into a 5 mL-reaction tube with 1.5 mL of TRIzol™ LS Reagent (Thermo Fisher Scientific, Waltham, MA). Total RNA was extracted following the procedural guidelines in the reagent user guide with the addition of three ethanol washes to remove excess salts prior to solubilization of RNA in nuclease-free water. Extracted RNA quality was checked using Tape Station (Agilent Technologies, Santa Clara, CA). Total RNA was normalized and complimentary DNA libraries were prepared using a Kapa mRNA Hyperprep kit without polyA enrichment step, cDNA libraries were sequenced on the Illumina NovaSeq S4 System (paired end, 150 bp) at the Bauer Core Facility at Harvard University.

### Procedural Controls.

We collected procedural negative controls that were carried through library preparation and sequencing. While sampling, we collected negative controls for the syringes and gavage needles used for calcifying fluid collections by using nuclease-free water and treating it as a true sample. We included RNA extraction negative controls with every batch. Additionally, we sequenced three replicates of a mock community (ZymoBIOMICS™ Microbial Community RNA Standard, Zymo Research, United States), with known theoretical relative abundances of 10 species, as a positive control.

### Shotgun Metatranscriptomic Sequence Analysis.

Paired-end reads were quality-filtered using Trim Galore v.0.6.10 (60) to trim, remove adapter content, and to ensure a minimum PHRED score of 30 and length of at least 120 bp. The quality of filtered reads was assessed with FastQC v.0.12.1 (61). SortMeRNA v.4.3.6 (62) was used to align quality-filtered reads against the SILVA SSU (16S,18S) and SILVA LSU (23S, 28S), to remove rRNA reads. We used splice-aware mapper STAR v.2.7.11b (63) to map the quality filtered, nonribosomal reads against the *C. virginica* transcriptome, downloaded from NCBI, to separate oyster from nonoyster reads.

### Oyster Transcriptome Analyses.

Oyster transcriptome annotations and assemblies were downloaded from NCBI. To complement NCBI’s annotations, we predicted open reading frames (ORFs) from the assemblies using Prodigal v.2.6.3 (64) and annotated using DIAMOND v.2.1.8 (65) in eggNOG-mapper v.2.1.11 (66). EggNOG-mapper uses orthologous groups and phylogenies from the eggNOG database to transfer functional information from fine-grained orthologs to the identified ORFs. The functional annotations per query provided by eggNOG-mapper include predicted protein name; KEGG pathways, modules, and orthologs (67); GO labels (68); EC numbers, BiGG reactions (69); CAZy terms (70); COG functional categories (71); eggNOG OGs; and text descriptions at all taxonomic levels. Sequence alignment map (SAM) files from STAR mapper were used to quantify oyster transcripts at the isoform level and normalized by gene length and sample sequencing depth as fragments per kilobase million (FPKM) using Salmon v.1.10.3 (72).

### Microbiome Metatranscriptome Analyses.

Nonoyster reads were used to assemble transcripts de novo, independently for each sample, with rnaSPAdes v.3.15.5 (73). The assembled transcripts were then aligned to the *C. virginica* genome using BLAST to detect residual oyster transcripts, which were filtered out using a Python script. The remaining nonoyster transcripts were used for downstream functional potential analyses. We predicted ORFs using Prodigal v.2.6.3 (64) and annotated using DIAMOND v.2.1.8 (65) in eggNOG-mapper v.2.1.11 (66). Quality-filtered nonoyster reads were then mapped to the ORFs using Bowtie2 v.2.3.2 (74) in very sensitive local mode to estimate abundance. Read counts of ORFs were normalized by gene length and sample sequencing depth as FPKM.

### Oyster Transcriptome Statistical Analyses.

Differential expression analysis of oyster transcripts was conducted using the DESeq2 v.1.40.2 R package. Transcripts were prefiltered to remove low-abundance features, retaining only those with at least five reads in at least two samples. This filtering reduced the dataset from 66,451 to 55,005 transcripts used in downstream analyses. Differential expression was tested using a multifactor design that included main effects of time point and treatment as well as their interaction (~Time point + Treatment + Time point:Treatment). Transcripts were considered significantly differentially expressed at an adjusted *P*-value threshold of <0.01, correcting for multiple testing using the Benjamini–Hochberg procedure. To investigate the expression of biomineralization-related genes, we curated a list of molluscan biomineralization toolkit genes based on functional categories defined by Zhuoqing et al. (32), including ion transporters, structural scaffold proteins, and regulators of mineral phase transformation. Transcripts were assigned to these categories using GO terms associated with their annotated functions. From the set of differentially expressed transcripts identified by DESeq2, we extracted those matching the biomineralization toolkit and visualized their log2 fold change across treatments and time points, grouped by functional role. GO enrichment analysis at the Molecular Function level was performed using the TopGO v.2.52.0 R package (75) on the set of significantly differentially expressed transcripts for each time point and treatment comparison.

### Microbiome Metatranscriptome Statistical Analyses.

Differential expression analyses of microbiome transcripts were conducted using the DESeq2 v.1.40.2 R package. A total of 3,810 KEGG Orthologs (KOs) were initially included. To reduce noise from low-abundance or rarely expressed genes, KOs with fewer than five total reads or appearing in fewer than two samples were removed, resulting in 2,644 KOs retained for downstream analysis. Differential expression was assessed using a multifactor design (~Time point + Treatment + Time point:Treatment). Features were considered significantly differentially expressed at an adjusted *P*-value threshold of 0.01, corrected for multiple testing using the Benjamini–Hochberg procedure.

To identify broader functional patterns, pathway enrichment analyses were performed using the MicrobiomeProfiler v1.11.0 R package (76). This analysis utilized KEGG KO annotations and DESeq2 output to identify KEGG pathways significantly enriched in differentially expressed genes for each time point and treatment comparison. Pathways with an adjusted *P*-value < 0.01 were considered significantly enriched.

To explore correlations between microbial gene expression and environmental variables, a separate series of DESeq2 regression models were run using single-factor designs: ~CF pH, ~CF DIC, ~tank water pH, and ~tank water DIC. These models used the same filtered KO dataset (2,644 KOs).

### Oyster and Microbiome Coexpression Analysis.

To investigate patterns of coexpression within and between the oyster host and its microbiome, we conducted independent weighted gene coexpression network analysis (WGCNA) using the WGCNA v.1.73 R package (77). Input data for each analysis consisted of log-transformed counts matrices (host or microbiome). For the host network, a soft-thresholding power of 5 was selected based on the scale-free topology fit index, while a power of 6 was used for the microbiome network. Network construction was performed using the blockwiseModules function with the following parameters: TOMType = “unsigned,” minModuleSize = 30, mergeCutHeight = 0.25, and pamRespectsDendro = FALSE. This approach identified 67 host modules and 19 microbiome modules of coexpressed genes.

For each dataset, module eigengenes (the first principal component of each module) were calculated and correlated with environmental parameters (seawater and calcifying-fluid pH and DIC) using the cor and corPvalueStudent functions in WGCNA R package. We then correlated host and microbiome module eigengenes to identify significantly covarying module pairs. Correlation results were visualized as a heatmap using the pheatmap R package v.1.0.12 (78) with modules annotated along the axes to indicate those significantly correlated with environmental parameters.

To further characterize these relationships, we quantified the percentage of host genes annotated to GO terms associated with host–microbe communication and symbiosis in modules that were significantly correlated with microbiome modules vs. those that were not. GO terms were classified as host–microbe communication if they fell under the parent categories symbiosis, encompassing mutualism through parasitism (GO:0044403), multiorganism signaling (GO:0051705), or response to symbiont (GO:0044416) within the Biological Process ontology (summarized in *SI Appendix*, Table S8). To test whether environmentally correlated host modules contained a higher percentage of host–microbe communication genes, we performed a Wilcoxon rank-sum test. Only host modules that were significantly correlated with microbiome modules were included in this analysis. Data normality was assessed using Shapiro–Wilk tests, which indicated that at least one group deviated from normality (*P* < 0.05). Because the data were nonnormal but similarly distributed across groups, we used the nonparametric Wilcoxon rank-sum test to evaluate group differences. For microbiome modules, since they were all significantly correlated with at least one host module, we next assessed the pathway completeness of KEGG Modules representing biochemical reactions that can alter carbonate chemistry (e.g., nitrogen and sulfur cycling, urease, and carbonic anhydrase) across all microbiome modules pooled. In addition, we performed pathway enrichment analyses to identify modules with significant overrepresentation of these KEGG processes using the KEGGREST R package v.1.40.1, with results summarized in *SI Appendix*, Table S10.

## Supplementary Material

Appendix 01 (PDF)

## Data Availability

Raw RNA-seq reads generated for this study are publicly available under NCBI BioProject accession PRJNA1313129 (79). Processed count matrices for both the oyster (*C. virginica*) host and its associated microbiome, along with all scripts used to generate and format these data, are available in the GitHub repository [https://github.com/Andrea-Unzueta-Martinez/oyster-host-microbiome-transcriptomics; (80)]. The repository contains the raw (unnormalized) host and microbiome count matrices, annotated Python scripts used for generating count tables and reformatting functional annotation files, and a detailed step-by-step pipeline describing the quality control, mapping, and quantification procedures.
