# Supplementary material for: Coexpression among eastern oyster host and microbiome genes suggests coordinated regulation of calcifying fluid chemistry
Source: Proc Natl Acad Sci U S A. 2026 Mar 10;123(11):e2521539123. doi: 10.1073/pnas.2521539123 (PMC12994172; doi:10.1073/pnas.2521539123)
Supplement: Supplementary file 1 — Appendix 01 (PDF) [file pnas.2521539123.sapp.pdf]

PNAS

Supporting Information for

Co-expression among eastern oyster host and microbiome genes suggests coordinated regulating of calcifying fluid chemistry

Andrea Unzueta-Martínez[1], Jennifer A. Delaney[1], Kate Morkeski[2], Abby Ross[3], Zhaohui Aleck Wang[2], Peter R. Girguis[1]

[1]Department of Organismic and Evolutionary Biology, Harvard University, Cambridge, MA, 02138
[2] Department of Marine Chemistry and Geochemistry, Woods Hole Oceanographic Institution, Woods Hole, MA 02543
[3]Department of Ecology and Evolutionary Biology and Institute of Arctic and Alpine Research (INSTAAR), University of Colorado Boulder, Boulder, CO 80303

Corresponding authors:
Andrea Unzueta-Martínez
Email: andrea_unzuetamartinez@fas.harvard.edu

Peter Girguis
Email: pgirguis@oeb.harvard.edu

This PDF file includes:

Tables S1 to S10
Figures S1 to S6

Table S1. Results of independent t-tests comparing calcifying fluid pH between control and tidal pH treatments at each timepoint across a 12-hour simulated tidal cycle. For each timepoint, the number of biological replicates (n = 4 per group), test statistic, degrees of freedom (df), raw p-value, and adjusted p-value (p.adj) are shown. P-values were adjusted using the Holm–Bonferroni method to account for multiple comparisons. A significant difference in calcifying fluid pH between treatments was observed only at the final timepoint (19:14; p.adj = 0.048). Significance levels are indicated in the final column (* = p.adj < 0.05; ns = not significant).

Timepoint	group1	group2	n1	n2	statistic	df	p	p.adj	p.adj.signif
6:25	Control	Tidal pH	4	4	-2.19	6	0.0713	0.119	ns
9:37	Control	Tidal pH	4	4	0.123	6	0.906	0.906	ns
12:50	Control	Tidal pH	4	4	2.58	6	0.042	0.105	ns
16:02	Control	Tidal pH	4	4	-0.255	6	0.807	0.906	ns
19:14	Control	Tidal pH	4	4	-3.74	6	0.0096	0.048	*

Table S2. (A) Results of a one-way ANOVA testing for differences in calcifying fluid pH across timepoints during the 12-hour simulated tidal cycle. A modest but statistically significant effect of timepoint was detected (*P* = 0.037), with a generalized eta-squared effect size (ges) of 0.474. (B) Results of the Tukey HSD post hoc test comparing calcifying fluid pH between timepoints. Pairwise comparisons show that calcifying fluid pH was generally stable throughout the experiment, with only one significant difference detected between 12:50 and 19:14 (p.adj = 0.0323). Confidence intervals and adjusted p-values (Holm–Bonferroni) are reported for all pairwise comparisons. Asterisks indicate statistically significant differences (* = p.adj < 0.05; ns = not significant).

A. ANOVA

Effect	DFn	DFd	F	p	p<.05	ges
Timepoint	4	15	3.377	0.037	*	0.474

B. Tukey HSD

Term	group1	group2	null.value	estimate	conf.low	conf.high	p.adj	p.adj.signif
Timepoint	6:25	9:37	0	0.0968	-0.254	0.447	0.909	ns
Timepoint	6:25	12:50	0	-0.132	-0.483	0.218	0.77	ns
Timepoint	6:25	16:02	0	-0.0663	-0.417	0.284	0.975	ns
Timepoint	6:25	19:14	0	0.245	-0.106	0.595	0.249	ns
Timepoint	9:37	12:50	0	-0.229	-0.58	0.121	0.304	ns
Timepoint	9:37	16:02	0	-0.163	-0.514	0.187	0.615	ns
Timepoint	9:37	19:14	0	0.148	-0.203	0.498	0.694	ns
Timepoint	12:50	16:02	0	0.066	-0.285	0.417	0.976	ns
Timepoint	12:50	19:14	0	0.377	0.0264	0.728	0.0323	*
Timepoint	16:02	19:14	0	0.311	-0.0396	0.662	0.0944	ns

Table S3. Results of independent t-tests comparing calcifying fluid dissolved inorganic carbon (DIC) between control and tidal pH treatments at each timepoint during the 12-hour tidal cycle experiment. Each test was performed independently (n = 4 per group per timepoint). No significant differences in DIC were detected between treatments at any timepoint after adjusting for multiple comparisons (Holm–Bonferroni method). Although a nominally significant difference was observed at 16:02 (P = 0.0314), it did not remain significant after adjustment (p.adj = 0.157). Significance levels are indicated in the final column (* = p.adj < 0.05; ns = not significant).

Timepoint	group1	group2	n1	n2	statistic	df	p	p.adj	p.adj.signif
6:25	Control	Tidal pH	4	4	2.2	6	0.0703	0.176	ns
9:37	Control	Tidal pH	4	4	0.406	6	0.698	0.698	ns
12:50	Control	Tidal pH	4	4	0.911	6	0.397	0.662	ns
16:02	Control	Tidal pH	4	4	-2.79	6	0.0314	0.157	ns
19:14	Control	Tidal pH	4	4	-0.579	6	0.583	0.698	ns

Table S4. (A) Results of a one-way ANOVA testing for differences in calcifying fluid dissolved inorganic carbon (DIC) across timepoints within the tidal pH treatment. A significant effect of timepoint was detected (*P* = 0.00073; generalized eta-squared [ges] = 0.701). (B) Tukey HSD post hoc pairwise comparisons between timepoints. Significant differences in DIC were observed between mid-day and late-day timepoints, with the 16:02 timepoint showing the strongest contrast: significantly higher DIC compared to 9:37 and 12:50, and significantly lower DIC than 19:14. Confidence intervals and adjusted p-values (Holm–Bonferroni) are reported. Significance levels are indicated in the final column (** = p.adj < 0.01; ns = not significant).

A. ANOVA

Effect	DFn	DFd	F	p	p<.05	ges
Timepoint	4	15	8.788	0.000733	*	0.701

B. Tukey HSD

Term	group1	group2	null.value	estimate	conf.low	conf.high	p.adj	p.adj.signif
Timepoint	6:25	9:37	0	486	-1247	2219	0.905	ns
Timepoint	6:25	12:50	0	153	-1581	1886	0.999	ns
Timepoint	6:25	16:02	0	2862	1129	4596	0.00106	**
Timepoint	6:25	19:14	0	441	-1293	2174	0.931	ns
Timepoint	9:37	12:50	0	-333	-2067	1400	0.974	ns
Timepoint	9:37	16:02	0	2376	643	4110	0.00554	**
Timepoint	9:37	19:14	0	-45.1	-1779	1688	1	ns
Timepoint	12:50	16:02	0	2709	976	4443	0.00177	**
Timepoint	12:50	19:14	0	288	-1445	2022	0.985	ns
Timepoint	16:02	19:14	0	-2421	-4155	-688	0.00474	**

Table S5. Summary of the seawater carbonate system over the course of the tidal simulation treatment. Average, standard deviation, range, and number of samples are presented for each sampling timepoint for the control and tidal pH treatments. Measured seawater parameters: Salinity, Temperature, pH, and dissolved inorganic carbon (DIC). Calculated parameters using 'seacarb' R package: Total alkalinity (TA), partial pressure of carbon dioxide (pCO_2), carbonate ion concentration (CO_3^{2-}), bicarbonate ion concentration (HCO_3^-), dissolved carbon dioxide concentration (CO_2), calcite saturation state ($\Omega_{CALCITE}$) and aragonite saturation state ($\Omega_{ARAGONITE}$)

Measured Parameters		Control treatment seawater					Tidal pH treatment seawater				
		6:25	9:37	12:50	16:02	19:14	6:25	9:37	12:50	16:02	19:14
Salinity (psu)	Average	35.16	34.80	34.71	34.88	34.93	33.96	32.24	32.78	32.72	32.74
	SD	1.12	0.17	0.06	0.19	0.12	1.74	4.23	4.14	4.16	4.04
	Range	34.6 - 36.2	34.5-34.9	34.6-34.7	34.6-35.1	34.7-35.0	31.3 - 34.9	25.8 - 34.5	26.5 - 34.9	26.4 - 35.0	26.6 - 34.8
	n	4	4	4	4	4	4	4	4	4	4
Temp (°C)	Average	18.40	17.70	17.60	17.80	17.70	18.06	17.38	17.63	17.50	18.12
	SD	0.12	0.30	0.30	0.40	0.30	0.50	0.15	0.25	0.33	0.22
	Range	18.1 - 18.4	17.3 - 18.0	17.2 - 18.0	17.2 - 18.1	17.2 - 18.1	17.5 - 18.7	17.3 - 17.6	17.3 - 17.8	17.1 - 17.8	17.8 - 18.3
	n	4	4	4	4	4	4	4	4	4	4
pH (NBS scale)	Average	7.83	7.74	7.65	7.64	7.65	7.87	7.24	6.93	7.48	7.65
	SD	0.03	0.03	0.04	0.02	0.02	0.01	0.10	0.07	0.04	0.07
	Range	7.79 - 7.86	7.70 - 7.77	7.61 - 7.69	7.63 - 7.68	7.62 - 7.68	7.86 - 7.88	7.11 - 7.35	6.85 - 7.00	7.42 - 7.52	7.61 - 7.75
	n	4	4	4	4	4	4	4	4	4	4
DIC (µmol/kg)	Average	2034.17	2003.07	1928.65	1896.28	1930.82	2009.03	2152.53	2194.43	2050.75	2022.88
	SD	109.02	130.05	102.32	31.04	127.76	126.68	117.13	75.89	113.44	119.68
	Range	1873 - 2110	1814 - 2111	1814 - 2051	1880 - 1942	1821 - 2084	1891 - 2148	1995 - 2276	2123 - 2295	1981 - 2218	1869 - 2161
	n	4	4	4	4	4	4	4	4	4	4
Calculated parameters											
TA (µmol/kg)	Average	2040.59	1900.76	1941.21	1867.81	1840.65	2068.61	1860.59	1874.32	2094.04	2013.93
	SD	95.00	125.60	117.85	91.65	27.83	127.72	97.15	117.09	78.92	110.16
	Range	1904 - 2110	1793 - 2046	1767 - 2031	1769 - 1978	1825 - 1882	1945 - 2209	1723 - 1952	1714 - 1994	2030 - 2203	1941 - 2176
	n	4	4	4	4	4	4	4	4	4	4
pCO_2 (ppm-v)	Average	640.15	750.28	968.25	942.90	907.46	592.62	2683.63	5586.42	1655.97	1032.72
	SD	70.97	70.20	142.16	94.72	49.79	42.83	693.66	781.35	116.54	148.20
	Range	540 - 704	687 - 842	823 - 1128	956 - 1039	842 - 958	543 - 637	2457 - 3688	4644 - 6554	1557 - 1824	825 - 1173
	n	4	4	4	4	4	4	4	4	4	4
CO_3^{2-} (µmol/kg)	Average	109.01	81.39	69.44	65.37	66.09	143.82	34.37	17.87	65.65	89.44
	SD	5.81	8.14	5.62	1.95	2.46	9.25	5.04	4.35	6.81	6.40
	Range	100 - 113	71.6 - 89.3	65.4 - 77.8	62.5 - 66.7	63.6 - 69.3	134 - 154	28.2 - 38.8	12.9 - 22.6	59.5 - 75.3	82.9 - 96.5
	n	4	4	4	4	4	4	4	4	4	4
HCO_3^- (µmol/kg]	Average	1766.06	1692.88	1764.83	1700.17	1670.66	1712.52	1774.88	1829.89	1933.37	1793.17
	SD	100.31	110.99	115.87	91.64	28.11	107.73	99.92	108.47	66.51	98.54
	Range	1617 - 1834	1596 - 1828	1598 - 1863	1597 - 1809	1649 - 1712	1613 - 1830	1640 - 1881	1682 - 1944	1869 - 2018	1733 - 1939
	n	4	4	4	4	4	4	4	4	4	4
CO_2 (µmol/kg)	Average	21.76	26.17	33.56	32.89	31.50	17.04	77.05	159.44	47.25	29.68
	SD	2.59	2.14	4.69	3.30	1.64	1.24	19.51	22.33	2.75	3.52
	Range	18.1 - 24.1	24.16 - 28.9	28.8 - 38.8	28.5 - 36.4	29 - 32	15.7 - 18.32	62.2 - 106	130 - 184	45.7 - 51.4	24.8 - 33.2
	n	4	4	4	4	4	4	4	4	4	4
$\Omega_{CALCITE}$	Average	2.60	1.95	1.66	1.56	1.58	3.49	0.85	0.44	1.61	2.19
	SD	0.13	0.20	0.13	0.04	0.06	0.19	0.14	0.10	0.15	0.19
	Range	2.4 - 2.7	1.71 - 2.14	1.54 - 1.86	1.5 - 1.59	1.52 - 1.66	3.33 - 3.72	0.68 - 0.99	0.33 - 0.54	1.44 - 1.81	2 - 2.38
	n	4	4	4	4	4	4	4	4	4	4
$\Omega_{ARAGONITE}$	Average	1.68	1.26	1.07	1.01	1.02	2.29	0.55	0.29	1.05	1.43
	SD	0.09	0.13	0.09	0.03	0.04	0.13	0.09	0.07	0.10	0.11
	Range	1.5 - 1.7	1.1 -1.4	1 - 1.2	0.97 - 1.02	0.98 - 1.07	2.17 - 2.44	0.44 - 0.63	0.22 - 0.36	0.95 - 1.19	1.32 - 1.53
	n	4	4	4	4	4	4	4	4	4	4

Table S6. Summary of the calcifying fluid carbonate system over the course of the tidal simulation treatment. Average, standard deviation, range, and number of samples are presented for each sampling timepoint for the control and tidal pH treatments. Measured calcifying fluid parameters: Salinity, Temperature, pH, and dissolved inorganic carbon (DIC). Calculated parameters using ‘seacarb’ R package: Total alkalinity (TA), partial pressure of carbon dioxide (pCO_2), carbonate ion concentration (CO_3^{2-}), bicarbonate ion concentration (HCO_3^-), dissolved carbon dioxide concentration (CO_2), calcite saturation state ($\Omega_{CALCITE}$) and aragonite saturation state ($\Omega_{ARAGONITE}$).

Measured Parameters		Control treatment calcifying fluid					Tidal pH treatment calcifying fluid				
		6:25	9:37	12:50	16:02	19:14	6:25	9:37	12:50	16:02	19:14
Salinity (psu)	Average	16.91	16.53	16.69	18.32	19.14	22.34	22.39	21.06	22.54	22.55
	SD	0.38	0.17	0.19	18.32	0.52	4.23	3.12	5.59	3.53	3.48
	Range	16.4 - 17.2	20.9 - 27.0	16.6 - 16.9	18.2 - 18.5	18.5 - 19.5	18.6 - 28.4	20.9 - 27.0	14.4 - 28.0	19.7 - 27.1	19.7 - 27.6
	n	4	4	4	4	4	4	4	4	4	4
Temp (°C)	Average	24.83	25.95	25.03	25.40	24.70	24.59	24.95	25.13	25.13	24.75
	SD	1.45	0.38	0.29	0.12	0.24	0.31	0.24	0.33	0.35	0.34
	Range	22.6 - 25.7	25.4 - 26.4	24.6 - 25.3	25.3 - 25.5	24.4 - 24.9	24.2 - 24.9	24.6 - 25.1	24.6 - 25.3	24.6 - 25.3	24.3 - 25.0
	n	4	4	4	4	4	4	4	4	4	4
pH (NBS scale)	Average	6.87	7.32	7.21	7.11	7.02	7.21	7.30	7.07	7.14	7.45
	SD	0.26	0.10	0.06	0.18	0.17	0.16	0.17	0.09	0.20	0.16
	Range	6.61 - 7.23	7.17 - 7.40	7.16 - 7.26	6.90 - 7.30	6.80 - 7.20	6.97 - 7.32	7.07 - 7.44	6.99 - 7.20	6.87 - 7.30	7.31 - 7.65
	n	4	4	4	4	4	4	4	4	4	4
DIC (umolkg-1)	Average	4247.06	3938.23	3669.84	3926.99	3415.04	3219.66	3705.69	3372.45	6081.90	3660.59
	SD	454.18	833.45	552.90	888.90	746.54	816.87	783.90	347.20	1260.13	401.24
	Range	3858 - 4848	3198 - 5026	3195 - 4468	3064 - 4699	2863 - 4517	4230 - 2495	3187 - 4861	3015 - 3785	4463 - 6846	3361 - 4225
	n	4	4	4	4	4	4	4	4	4	4
Calculated parameters											
TA (µmol/kg)	Average	3764.87	3764.87	3525.18	3710.69	3180.47	3103.13	3633.92	3186.92	5816.85	3658.74
	SD	231.22	231.22	516.99	841.00	731.09	764.07	807.62	329.03	1307.94	411.19
	Range	3469 - 4017	3469 - 4017	3049 - 4259	2811 - 4510	2663 - 42249	2445 - 4097	3168 - 4839	2835 - 3549	4272 - 7108	3318 - 4245
	n	4	4	4	4	4	4	4	4	4	4
pCO_2 (ppm-v)	Average	16278.53	5308.37	6117.60	8501.91	8597.17	5477.89	4858.63	7317.20	11524.54	3473.52
	SD	8829.57	1660.11	1401.53	3615.62	2968.41	2754.41	1715.56	1219.44	4739.00	1108.50
	Range	6105 - 27662	3476 - 7434	5148 - 8159	4252 - 13019	5233 - 11944	3125 - 8943	3191 - 7260	6102 - 8994	8474 - 18594	1935 - 4386
	n	4	4	4	4	4	4	4	4	4	4
CO_3^{2-} (µmol/kg)	Average	22.03	56.43	39.27	37.06	26.20	40.29	62.97	29.85	72.75	86.54
	SD	14.47	18.35	5.92	15.91	11.32	10.29	28.59	2.85	35.61	30.29
	Range	11.9 - 43.5	42 - 82.7	30.5 - 43.5	18.1 - 52.9	13.4 - 36	30.2 - 54.6	36 - 102.5	27.3 - 33.7	34.1 - 108	51.7 - 116
	n	4	4	4	4	4	4	4	4	4	4
HCO_3^- (µmol/kg	Average	3717.51	3720.34	3440.11	3630.42	3122.93	3013.27	3496.04	3120.91	5662.81	3468.65
	SD	239.63	790.29	510.31	825.58	714.18	752.03	756.31	325.89	1245.21	386.63
	Range	3436 - 3992	3037 - 4771	2982 - 4170	2771 - 4397	2619 - 4171	2361 - 3979	3026 - 4619	2772 - 3483	4159 - 6881	3185 - 4015
	n	4	4	4	4	4	4	4	4	4	4
CO_2 (µmol/kg)	Average	507.52	161.46	190.46	259.51	265.91	166.12	146.67	221.71	346.33	105.41
	SD	268.27	50.80	45.01	109.73	90.31	80.18	50.16	35.09	139.85	33.12
	Range	188 - 844	105 - 225	160 - 256	130 - 396	162 - 367	97 - 265	96 - 216	191 - 272	252 - 554	59 - 130
	n	4	4	4	4	4	4	4	4	4	4
$\Omega_{CALCITE}$	Average	0.61	1.58	1.09	1.02	0.71	1.07	1.68	0.81	1.95	2.30
	SD	0.41	0.51	0.16	0.44	0.31	0.29	0.79	0.12	0.99	0.82
	Range	0.33 - 1.22	1.17 - 2.31	0.85 - 1.21	0.49 - 1.46	0.36 - 0.99	0.76 - 1.47	0.94 - 2.76	0.69 - 0.96	0.89 - 2.94	1.39 - 3.11
	n	4	4	4	4	4	4	4	4	4	4
$\Omega_{ARAGONITE}$	Average	0.37	0.96	0.67	0.63	0.44	0.67	1.05	0.50	1.22	1.44
	SD	0.25	0.31	0.10	0.27	0.19	0.18	0.48	0.06	0.61	0.51
	Range	0.20 - 0.74	0.72 - 1.41	0.52 - 0.74	0.31 - 0.89	0.22 - 0.61	0.49 - 0.92	0.61 - 1.72	0.45 - 0.58	0.57 - 1.82	0.86 - 1.95
	n	4	4	4	4	4	4	4	4	4	4

Table S7. Molluscan biomineralization toolkit. Gene families included in the molluscan biomineralization toolkit, based on Zhuoquing et al. (1). Each row represents a functionally distinct protein family implicated in shell formation and carbonate chemistry regulation. The table includes the representative GO term, family name, functional description, number of transcripts in our dataset annotated to that family, and primary references. Functional roles span matrix scaffolding, ion transport, crystal nucleation, and carbonate conversion. GO annotations are based on transcript-level mappings, and transcript counts reflect the number of oyster calcifying fluid transcripts matched to each family.

Representative GO Term	Protein family	Function	Number of transcripts in our study annotated to each protein family	Primary Reference(s)
GO:0006030	Chitin synthase	Synthesizes chitin chains for the shell framework	5	2,3
GO:0004568	Chitinase	Hydrolyzes chitin; regulates chitin remodeling	1	4,5
GO:0005576	Shematrin	Gly-rich domain protein; structural scaffold	138	6,7
GO:0051213	KRMP	Lys-rich matrix protein; inhibits CaCO3 precipitation	0	8,9
GO:0004867	Prisilkin-39	Gly/Tyr/Ser-rich; inhibits aragonite deposition	0	10
GO:0005515	Prismalin-14	Binds chitin and CaCO3; dual functionality	683	11,12
GO:0005509	C1q domain proteins	Matrix structuring; responds to pathogens	122	13,14
GO:0007155	FN3 domain proteins	Mineral layer arrangement; adhesion	97	15,16
GO:0030414	TIMP	Inhibits proteases; protects matrix	16	17,18
GO:0005388	PMCA	Ca2+ transport; high affinity, low capacity	4	19
GO:0005389	NCX	Na+/Ca2+ exchanger; bidirectional	0	19,20
GO:0005245	VDCC	Voltage-dependent Ca2+ channel	3	21,22
GO:0015701	Bicarbonate transporter (SLC4)	HCO3- supply for calcification	9	23,24,20
GO:0005391	Na+/K+-ATPase	Maintains Na+ gradient for other ion transport	0	20,25
GO:0015385	H+-ATPase / NHE / VGHC	Removes protons; regulates pH	185	26
GO:0004089	Nacrein	Carbonic anhydrase + Gly-X-Asn domain; inhibits CaCO3 nucleation	0	27,28
GO:0005509	MSP-1	Asp-rich; template for calcite nucleation	44	29
GO:0005509	Aspein	Highly acidic; promotes calcite nucleation	0	30
GO:0005509	PfY2	Asp-rich; inhibits CaCO3 growth	320	31
GO:0004867	PNU7	Inhibits calcite; stabilizes vaterite	0	32
GO:0005509	Pif80	Asn-rich; shapes aragonite tablets	56	33,34
GO:0005509	PU14	Gln-rich; regulates calcite morphology	958	35
GO:0005509	Tyrosinase	Crosslinks matrix proteins; inhibits CaCO3 precipitation	0	36,37
GO:0008270	Carbonic anhydrase	Catalyzes CO2/HCO3- conversion; regulates pH	41	38

Table S8. Summary of Gene Ontology (GO) terms related to host-microbe communication. List of GO terms associated with host-microbe and symbiosis-related communication functions identified in oyster host modules. The table includes the GO term ID, term description, number of genes annotated to each term, and the modules in which these genes occur. GO terms were classified as ‘host-microbe communication’ if they fell under the parent categories ‘symbiosis, encompassing mutualism through parasitism’ (GO:0044403), ‘multi-organism signaling’ (GO:0051705), or ‘response to symbiont’ (GO:0044416) within the Biological Process ontology.

GOs	Term	n_modules	n_genes
GO:0051707	response to other organism	54	772
GO:0009617	response to bacterium	51	576
GO:0098542	defense response to other organism	49	439
GO:0042742	defense response to bacterium	42	277
GO:0002237	response to molecule of bacterial origin	43	236
GO:0032496	response to lipopolysaccharide	41	209
GO:0009615	response to virus	41	194
GO:0016032	viral process	41	170
GO:0051607	defense response to virus	38	140
GO:0050688	regulation of defense response to virus	35	127
GO:0050792	regulation of viral process	32	121
GO:0002221	pattern recognition receptor signaling pathway	28	117
GO:0050829	defense response to Gram-negative bacterium	31	117
GO:0042579	microbody	35	116
GO:0071219	cellular response to molecule of bacterial origin	36	112
GO:0034121	regulation of toll-like receptor signaling pathway	29	109
GO:0019058	viral life cycle	33	99
GO:1903900	regulation of viral life cycle	29	98
GO:0051701	biological process involved in interaction with host	37	96
GO:0071222	cellular response to lipopolysaccharide	33	96
GO:0009620	response to fungus	26	95
GO:0002224	toll-like receptor signaling pathway	28	92
GO:0050830	defense response to Gram-positive bacterium	26	90
GO:0002759	regulation of antimicrobial humoral response	27	81
GO:0002784	regulation of antimicrobial peptide production	26	78
GO:1900424	regulation of defense response to bacterium	25	77
GO:0050832	defense response to fungus	20	73
GO:0048525	negative regulation of viral process	27	72
GO:0002760	positive regulation of antimicrobial humoral response	23	67
GO:0002225	positive regulation of antimicrobial peptide production	23	66
GO:1903901	negative regulation of viral life cycle	25	64
GO:0050691	regulation of defense response to virus by host	27	63
GO:0019730	antimicrobial humoral response	25	58
GO:0034123	positive regulation of toll-like receptor signaling pathway	24	57
GO:0002805	regulation of antimicrobial peptide biosynthetic process	24	56
GO:0002230	positive regulation of defense response to virus by host	26	55
GO:1900426	positive regulation of defense response to bacterium	23	52
GO:0002786	regulation of antibacterial peptide production	23	51
GO:0002808	regulation of antibacterial peptide biosynthetic process	23	51
GO:0002807	positive regulation of antimicrobial peptide biosynthetic process	22	49
GO:0034122	negative regulation of toll-like receptor signaling pathway	21	49
GO:0048524	positive regulation of viral process	20	49
GO:0002813	biosynthetic process of antibacterial peptides active against Gram-negative bacteria	20	47
GO:0002803	positive regulation of antibacterial peptide production	22	46
GO:0006963	positive regulation of antibacterial peptide biosynthetic process	22	46
GO:0061057	peptidoglycan recognition protein signaling pathway	21	46
GO:0031903	microbody membrane	24	44
GO:0002756	MyD88-independent toll-like receptor signaling pathway	22	43
GO:0006964	biosynthetic process of antibacterial peptides active against Gram-negative bacteria	20	43
GO:0019048	modulation by virus of host process	21	42
GO:0019079	viral genome replication	19	42
GO:0039531	regulation of viral-induced cytoplasmic pattern recognition receptor signaling pathway	22	42
GO:0044003	modulation by symbiont of host process	21	42
GO:0035666	TRIF-dependent toll-like receptor signaling pathway	22	41
GO:0031907	microbody lumen	18	36
GO:0045069	regulation of viral genome replication	17	36
GO:0046596	regulation of viral entry into host cell	19	36
GO:0051702	biological process involved in interaction with symbiont	18	35
GO:0051851	modulation by host of symbiont process	17	33
GO:0046718	viral entry into host cell	18	32

GO:1903902	positive regulation of viral life cycle	15	32
GO:0060314	regulation of ryanodine-sensitive calcium-release channel activity	17	31
GO:0098586	cellular response to virus	19	31
GO:0046597	negative regulation of viral entry into host cell	17	30
GO:0016045	detection of bacterium	13	24
GO:0002753	cytosolic pattern recognition receptor signaling pathway	15	23
GO:0019054	modulation by virus of host cellular process	14	23
GO:0034142	toll-like receptor 4 signaling pathway	14	23
GO:0044068	modulation by symbiont of host cellular process	14	23
GO:0050687	negative regulation of defense response to virus	16	22
GO:0034138	toll-like receptor 3 signaling pathway	14	21
GO:0019080	viral gene expression	14	20
GO:0019081	viral translation	14	20
GO:0019731	antibacterial humoral response	14	20
GO:0034162	toll-like receptor 9 signaling pathway	13	20
GO:0046794	transport of virus	13	20
GO:2000535	regulation of entry of bacterium into host cell	12	20
GO:0038187	pattern recognition receptor activity	9	19
GO:0045071	negative regulation of viral genome replication	13	19
GO:0039694	viral RNA genome replication	13	18
GO:1900150	regulation of defense response to fungus	11	18
GO:0002755	MyD88-dependent toll-like receptor signaling pathway	11	17
GO:0045070	positive regulation of viral genome replication	10	17
GO:0046782	regulation of viral transcription	10	17
GO:0061844	antimicrobial humoral immune response mediated by antimicrobial peptide	10	17
GO:0075522	IRES-dependent viral translational initiation	12	17
GO:0031663	lipopolysaccharide-mediated signaling pathway	10	16
GO:0033268	node of Ranvier	10	16
GO:0034124	regulation of MyD88-dependent toll-like receptor signaling pathway	12	16
GO:0075733	intracellular transport of virus	9	16
GO:0001618	virus receptor activity	8	15
GO:0032490	detection of molecule of bacterial origin	11	15
GO:0033270	paranode region of axon	8	15
GO:0035325	Toll-like receptor binding	11	15
GO:0046755	viral budding	9	15
GO:0060992	response to fungicide	11	15
GO:1900425	negative regulation of defense response to bacterium	12	15
GO:0001530	lipopolysaccharide binding	11	14
GO:0034134	toll-like receptor 2 signaling pathway	10	13
GO:0034143	regulation of toll-like receptor 4 signaling pathway	10	13
GO:0034154	toll-like receptor 7 signaling pathway	11	13
GO:0043921	modulation by host of viral transcription	9	13
GO:0052472	modulation by host of symbiont transcription	9	13
GO:0071205	protein localization to juxtaparanode region of axon	9	13
GO:0009597	detection of virus	8	12
GO:0030913	paranodal junction assembly	8	12
GO:0039532	viral-induced cytoplasmic pattern recognition receptor signaling pathway	11	12
GO:0039702	viral budding via host ESCRT complex	9	12
GO:0044224	juxtaparanode region of axon	8	12
GO:0044788	modulation by host of viral process	8	12
GO:0050434	positive regulation of viral transcription	7	12
GO:0086016	AV node cell action potential	10	12
GO:0086027	AV node cell to bundle of His cell signaling	10	12
GO:0086045	membrane depolarization during AV node cell action potential	10	12
GO:0086067	AV node cell to bundle of His cell communication	10	12
GO:0008348	negative regulation of antimicrobial humoral response	9	11
GO:0031664	regulation of lipopolysaccharide-mediated signaling pathway	9	11
GO:0034126	positive regulation of MyD88-dependent toll-like receptor signaling pathway	9	11
GO:0034127	regulation of MyD88-independent toll-like receptor signaling pathway	8	11
GO:0034128	negative regulation of MyD88-independent toll-like receptor signaling pathway	8	11
GO:0034135	regulation of toll-like receptor 2 signaling pathway	10	11
GO:0035635	entry of bacterium into host cell	9	11
GO:0002175	protein localization to paranode region of axon	7	10
GO:0016046	detection of fungus	5	10
GO:0034139	regulation of toll-like receptor 3 signaling pathway	9	10
GO:0031665	negative regulation of lipopolysaccharide-mediated signaling pathway	8	9
GO:0032493	response to bacterial lipoprotein	8	9
GO:0032494	response to peptidoglycan	7	9
GO:0034136	negative regulation of toll-like receptor 2 signaling pathway	8	9
GO:0034141	positive regulation of toll-like receptor 3 signaling pathway	9	9
GO:0035663	Toll-like receptor 2 binding	8	9
GO:0042494	detection of bacterial lipoprotein	8	9
GO:0043923	positive regulation by host of viral transcription	7	9
GO:0048535	lymph node development	6	9

GO:0061196	fungiform papilla development	7	9
GO:0070339	response to bacterial lipopeptide	8	9
GO:0070340	detection of bacterial lipopeptide	8	9
GO:0070891	lipoteichoic acid binding	7	9
GO:0071220	cellular response to bacterial lipoprotein	8	9
GO:0071221	cellular response to bacterial lipopeptide	8	9
GO:0002785	negative regulation of antimicrobial peptide production	8	8
GO:0034137	positive regulation of toll-like receptor 2 signaling pathway	8	8
GO:0034145	positive regulation of toll-like receptor 4 signaling pathway	7	8
GO:0035355	Toll-like receptor 2-Toll-like receptor 6 protein complex	7	8
GO:0038124	toll-like receptor TLR6:TLR2 signaling pathway	7	8
GO:0042496	detection of diacyl bacterial lipopeptide	7	8
GO:0042834	peptidoglycan binding	6	8
GO:0044827	modulation by host of viral genome replication	6	8
GO:0061197	fungiform papilla morphogenesis	6	8
GO:0071724	response to diacyl bacterial lipopeptide	7	8
GO:0071726	cellular response to diacyl bacterial lipopeptide	7	8
GO:0002752	cell surface pattern recognition receptor signaling pathway	5	7
GO:0019050	suppression by virus of host apoptotic process	7	7
GO:0035354	Toll-like receptor 1-Toll-like receptor 2 protein complex	7	7
GO:0038123	toll-like receptor TLR1:TLR2 signaling pathway	7	7
GO:0039506	modulation by virus of host molecular function	7	7
GO:0039507	suppression by virus of host molecular function	7	7
GO:0039513	suppression by virus of host catalytic activity	7	7
GO:0039516	modulation by virus of host catalytic activity	7	7
GO:0039526	modulation by virus of host apoptotic process	7	7
GO:0042495	detection of triacyl bacterial lipopeptide	7	7
GO:0044794	positive regulation by host of viral process	4	7
GO:0052040	modulation by symbiont of host programmed cell death	7	7
GO:0052055	modulation by symbiont of host molecular function	7	7
GO:0052148	modulation by symbiont of host catalytic activity	7	7
GO:0052150	modulation by symbiont of host apoptotic process	7	7
GO:0071725	response to triacyl bacterial lipopeptide	7	7
GO:0071727	cellular response to triacyl bacterial lipopeptide	7	7
GO:1990227	paranodal junction maintenance	5	7
GO:0000270	peptidoglycan metabolic process	5	6
GO:0002788	regulation of antifungal peptide production	4	6
GO:0002804	positive regulation of antifungal peptide production	4	6
GO:0009253	peptidoglycan catabolic process	5	6
GO:0019042	viral latency	3	6
GO:0019732	antifungal humoral response	4	6
GO:0033010	paranodal junction	6	6
GO:0039519	modulation by virus of host autophagy	6	6
GO:0044793	negative regulation by host of viral process	6	6
GO:0061058	regulation of peptidoglycan recognition protein signaling pathway	6	6
GO:0086026	atrial cardiac muscle cell to AV node cell signaling	6	6
GO:0086066	atrial cardiac muscle cell to AV node cell communication	6	6
GO:0002787	negative regulation of antibacterial peptide production	5	5

GO:0002806	negative regulation of antimicrobial peptide biosynthetic process	5	5
GO:0002809	negative regulation of antibacterial peptide biosynthetic process	5	5
GO:0005713	recombination nodule	5	5
GO:0019043	establishment of viral latency	3	5
GO:0019075	virus maturation	5	5
GO:0032897	negative regulation of viral transcription	5	5
GO:0034144	negative regulation of toll-like receptor 4 signaling pathway	5	5
GO:0043152	induction of bacterial agglutination	4	5
GO:0044828	negative regulation by host of viral genome replication	5	5
GO:0061060	negative regulation of peptidoglycan recognition protein signaling pathway	5	5
GO:0061783	peptidoglycan muralytic activity	3	5
GO:0070391	response to lipoteichoic acid	3	5
GO:0071223	cellular response to lipoteichoic acid	3	5
GO:0071852	fungal-type cell wall organization or biogenesis	5	5
GO:0075071	modulation by symbiont of host autophagy	5	5
GO:0075713	establishment of integrated proviral latency	3	5
GO:0000324	fungal-type vacuole	4	4
GO:0002810	regulation of antifungal peptide biosynthetic process	2	4
GO:0002814	biosynthetic process of antibacterial peptides active against Gram-negative bacteria	4	4
GO:0006967	positive regulation of antifungal peptide biosynthetic process	2	4
GO:0019064	fusion of virus membrane with host plasma membrane	4	4
GO:0019065	receptor-mediated endocytosis of virus by host cell	4	4
GO:0031505	fungal-type cell wall organization	4	4
GO:0034163	regulation of toll-like receptor 9 signaling pathway	4	4
GO:0034166	toll-like receptor 10 signaling pathway	4	4
GO:0039663	membrane fusion involved in viral entry into host cell	4	4
GO:0043922	negative regulation by host of viral transcription	4	4
GO:0044829	positive regulation by host of viral genome replication	3	4
GO:0046598	positive regulation of viral entry into host cell	4	4
GO:0060316	positive regulation of ryanodine-sensitive calcium-release channel activity	4	4
**GO:0061198**	**fungiform papilla formation**	**3**	**4**
GO:0075509	endocytosis involved in viral entry into host cell	4	4
GO:1902097	r transcription from RNA polymerase II promoter involved in defense response to Gram-negative bacterium	3	4
GO:0000329	fungal-type vacuole membrane	3	3
GO:0002238	response to molecule of fungal origin	3	3
GO:0009272	fungal-type cell wall biogenesis	3	3
GO:0015920	lipopolysaccharide transport	2	3
GO:0016019	peptidoglycan immune receptor activity	2	3
GO:0019072	viral genome packaging	3	3
GO:0019074	viral RNA genome packaging	3	3
GO:0034125	negative regulation of MyD88-dependent toll-like receptor signaling pathway	3	3
GO:0039520	induction by virus of host autophagy	3	3
GO:0039521	suppression by virus of host autophagy	3	3
GO:0039689	negative stranded viral RNA replication	3	3
GO:0044830	modulation by host of viral RNA genome replication	2	3
GO:0046719	regulation by virus of viral protein levels in host cell	2	3
GO:0046784	viral mRNA export from host cell nucleus	2	3
GO:0060315	negative regulation of ryanodine-sensitive calcium-release channel activity	3	3
GO:0071206	establishment of protein localization to juxtaparanode region of axon	2	3
GO:1903772	regulation of viral budding via host ESCRT complex	3	3
GO:1903774	positive regulation of viral budding via host ESCRT complex	3	3
GO:0002775	antimicrobial peptide production	2	2
GO:0002781	antifungal peptide production	2	2
GO:0002816	biosynthetic process of antibacterial peptides active against Gram-positive bacteria	1	2
GO:0005715	late recombination nodule	2	2
GO:0006965	regulation of biosynthetic process of antibacterial peptides active against Gram-positive bacteria	1	2
GO:0019087	transformation of host cell by virus	2	2
GO:0031666	positive regulation of lipopolysaccharide-mediated signaling pathway	2	2
GO:0034164	negative regulation of toll-like receptor 9 signaling pathway	2	2
GO:0035668	TRAM-dependent toll-like receptor signaling pathway	2	2
GO:0035669	TRAM-dependent toll-like receptor 4 signaling pathway	2	2

GO:0038092	nodal signaling pathway	2	2
GO:0039656	modulation by virus of host gene expression	2	2
GO:0043590	bacterial nucleoid	2	2
GO:0044790	suppression of viral release by host	2	2
GO:0046725	negative regulation by virus of viral protein levels in host cell	2	2
GO:0071940	fungal-type cell wall assembly	2	2
GO:1900107	regulation of nodal signaling pathway	2	2
GO:1900145	regulation of nodal signaling pathway involved in determination of left/right asymmetry	2	2
GO:1900175	nodal signaling pathway involved in determination of lateral mesoderm left/right asymmetry	2	2
GO:0003127	detection of nodal flow	1	1
GO:0003163	sinoatrial node development	1	1
GO:0009277	fungal-type cell wall	1	1
GO:0009609	response to symbiotic bacterium	1	1
GO:0019045	latent virus replication	1	1
GO:0019046	release from viral latency	1	1
GO:0019049	mitigation of host antiviral defense response	1	1
GO:0019056	modulation by virus of host transcription	1	1
GO:0019076	viral release from host cell	1	1
GO:0032499	detection of peptidoglycan	1	1
GO:0032995	regulation of fungal-type cell wall biogenesis	1	1
GO:0034140	negative regulation of toll-like receptor 3 signaling pathway	1	1
GO:0034165	positive regulation of toll-like receptor 9 signaling pathway	1	1
GO:0034178	toll-like receptor 13 signaling pathway	1	1
GO:0035660	MyD88-dependent toll-like receptor 4 signaling pathway	1	1
GO:0038100	nodal binding	1	1
GO:0045091	regulation of single stranded viral RNA replication via double stranded DNA intermediate	1	1
GO:0045870	single stranded viral RNA replication via double stranded DNA intermediate	1	1
GO:0046696	lipopolysaccharide receptor complex	1	1
GO:0046726	positive regulation by virus of viral protein levels in host cell	1	1
GO:0046745	viral capsid secondary envelopment	1	1
GO:0046778	modification by virus of host mRNA processing	1	1
GO:0052026	modulation by symbiont of host transcription	1	1
GO:0060237	regulation of fungal-type cell wall organization	1	1
GO:0060921	sinoatrial node cell differentiation	1	1
GO:0060931	sinoatrial node cell development	1	1
GO:0061059	positive regulation of peptidoglycan recognition protein signaling pathway	1	1
GO:0062040	fungal biofilm matrix	1	1
GO:0071226	cellular response to molecule of fungal origin	1	1
GO:0071341	medial cortical node	1	1
GO:0072573	tolerance induction to lipopolysaccharide	1	1
GO:0075525	viral translational termination-reinitiation	1	1
GO:0086015	SA node cell action potential	1	1
GO:0086018	SA node cell to atrial cardiac muscle cell signaling	1	1
GO:0086041	voltage-gated potassium channel activity involved in SA node cell action potential depolarization	1	1
GO:0086046	membrane depolarization during SA node cell action potential	1	1
GO:0086070	SA node cell to atrial cardiac muscle cell communication	1	1
GO:1900224	nodal signaling pathway involved in determination of lateral mesoderm left/right asymmetry	1	1
GO:1904187	regulation of transformation of host cell by virus	1	1
GO:1904188	negative regulation of transformation of host cell by virus	1	1

Table S9. Results of the Wilcoxon rank-sum test comparing the percentage of neuro-immune genes between environmentally correlated and non-correlated host modules. Only host modules that were significantly correlated with microbiome modules were included in this analysis. Data normality was evaluated using Shapiro-Wilk tests, which indicated that at least one group deviated from normality ($P < 0.05$). Because the data were non-normal but similarly distributed across groups, a non-parametric Wilcoxon rank-sum test was used to assess group differences.

Comparison	Subset included	n_1	n_2	Test statistic (W)	p-value
Env-sig vs. Not env-sig	Host modules correlated with microbiome modules	17	42	475.5	0.048

Table S10. Results of pathway enrichment analysis for microbiome modules, identifying KEGG biochemical reactions that can alter carbonate chemistry (nitrogen and sulfur cycling, urease, and carbonic anhydrase). Enrichment analyses were performed using the KEGGREST R package (v.1.40.1). Pathway completeness (%) represents the proportion of genes present in the pooled microbiome dataset. Enriched modules are also highlighted in Fig. 4C with stars.

Microbiome Module	KEGG Pathway	Gene Ratio	Background	Gene Count	p-value	Adjusted p-value (FDR)
ME4	M00529 Denitrification, nitrate → nitrogen	5/43	9/486	5	4.17×10^{-4}	0.0025
ME1	M00531 Assimilatory sulfate reduction, sulfate → H_2S	29/157	60/486	29	4.27×10^{-3}	0.0256
ME10	M00595 Thiosulfate oxidation by SOX complex, thiosulfate → sulfate	5/43	7/486	2	6.11×10^{-3}	0.0122

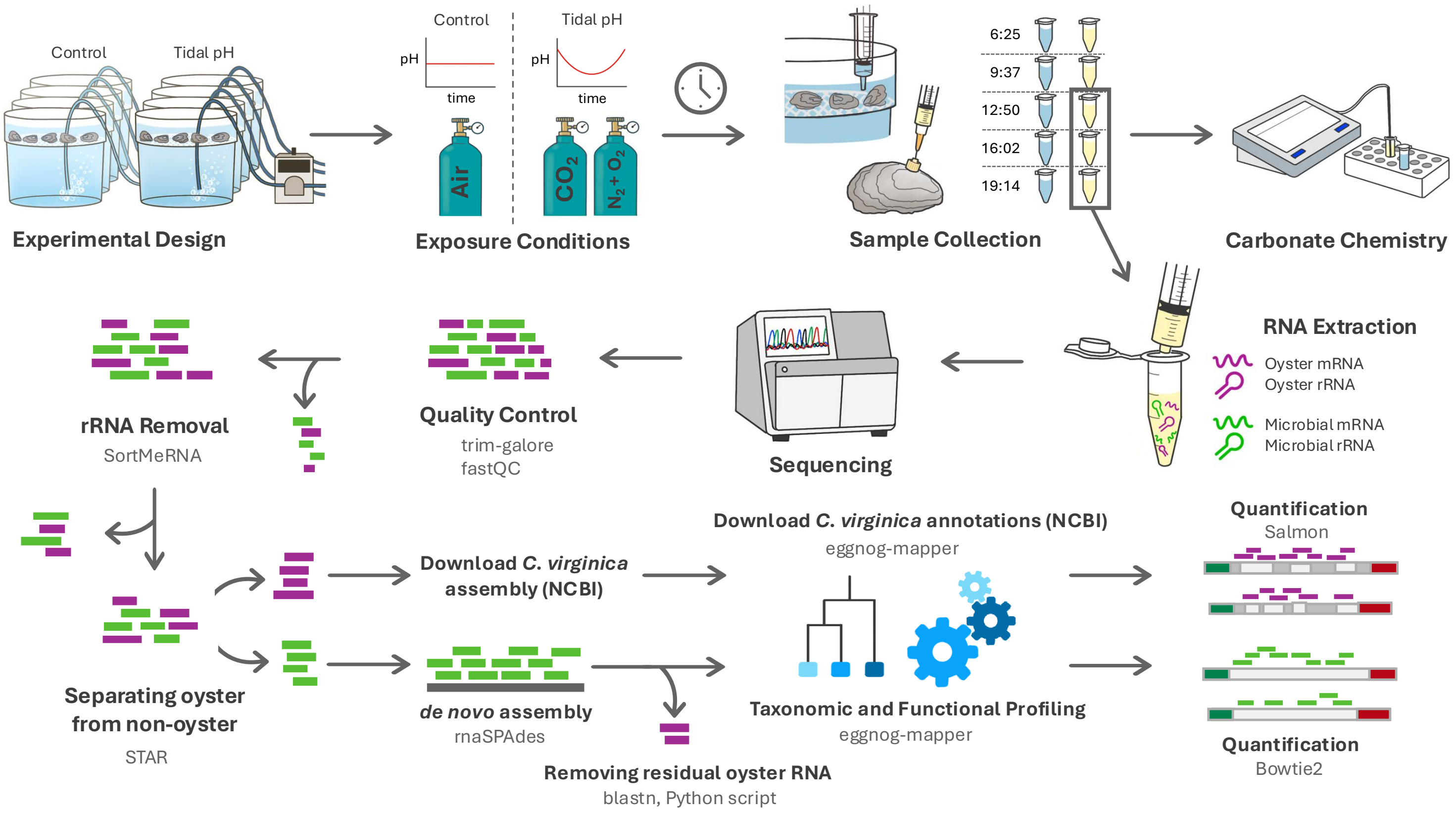

Figure S1. Summary of experimental design and metatranscriptomics workflow. To understand how the oyster and microbiomes respond to simulated tidal pH changes, we designed treatment that simulated changes in tidal cycle carbonate chemistry by using compressed gasses and bubbling them into seawater tanks. Oysters were exposed to either tidal or control conditions for 12 hours and 25 minutes (1 tidal cycle). Oyster calcifying fluids and seawater were collected from each of the five tanks every three hours and 12 minutes for carbonate chemistry analyses. We extracted and sequenced the total RNA from exposure and control calcifying fluid samples from the last three timepoints. The metatranscriptomics workflow consisted of pre-processing sequencing data to ensure quality, removal of rRNA reads, separation of host and microbiome reads. Host reads were annotated by querying reads against reference databases. Microbiome reads were *de novo* assembled into longer contiguous transcripts and again mapped against the host genome to removed remaining host transcripts before annotating their function and taxonomy against reference databases. Both host and microbiome transcript abundances were estimated using mapping tools. Annotations and counts estimates were used to conduct statistical analyses to associate expression information with sample timepoint and chemistry.

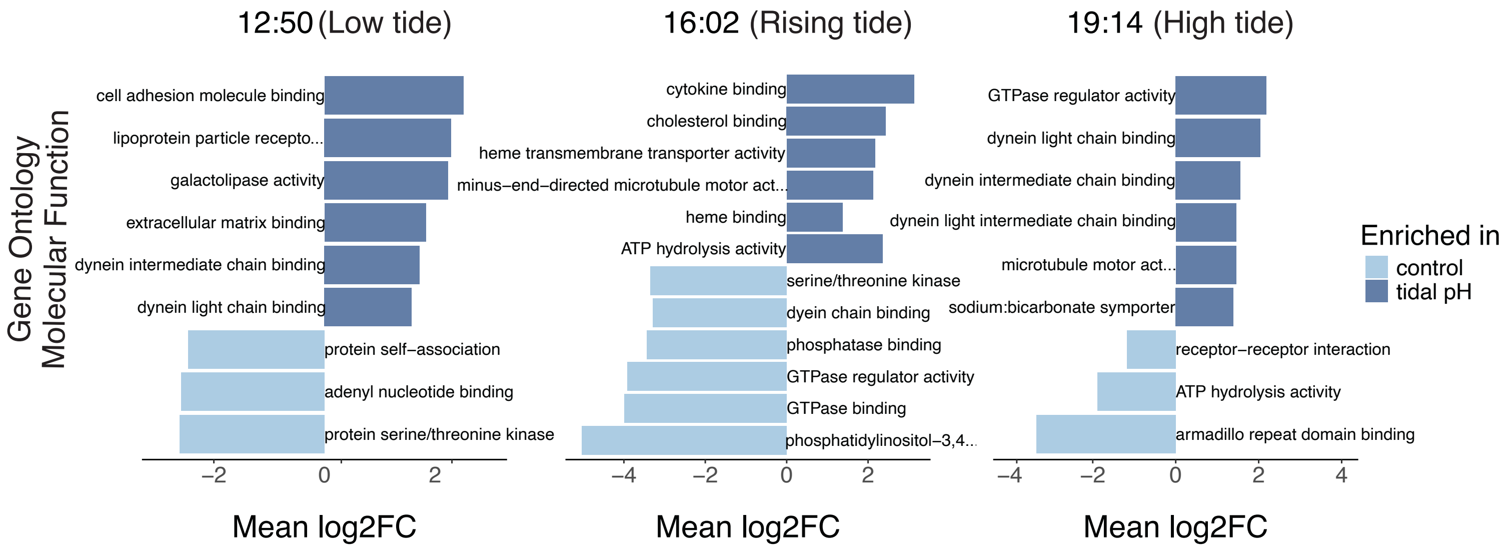

Figure S2. Gene Ontology (GO) enrichment analysis at the Molecular Function level for differentially expressed oyster transcripts across tidal cycle timepoints. GO terms were identified using the TopGO package (Fisher's exact test, $P < 0.05$) based on transcriptomic comparisons between tidal pH and control treatments at 12:50 (low tide), 16:02 (rising tide), and 19:14 (high tide). Bars represent the mean log2 fold change of all transcripts annotated to each significantly enriched GO term. GO terms enriched in the control treatment are shown in dark blue, while those enriched in the tidal pH treatment are shown in light blue. Functional enrichment patterns shift across timepoints, with structural and extracellular matrix-related functions enriched in tidal pH at 12:50, and more regulatory and ion transport-related functions enriched at later timepoints, potentially reflecting dynamic physiological responses to fluctuating external pH.

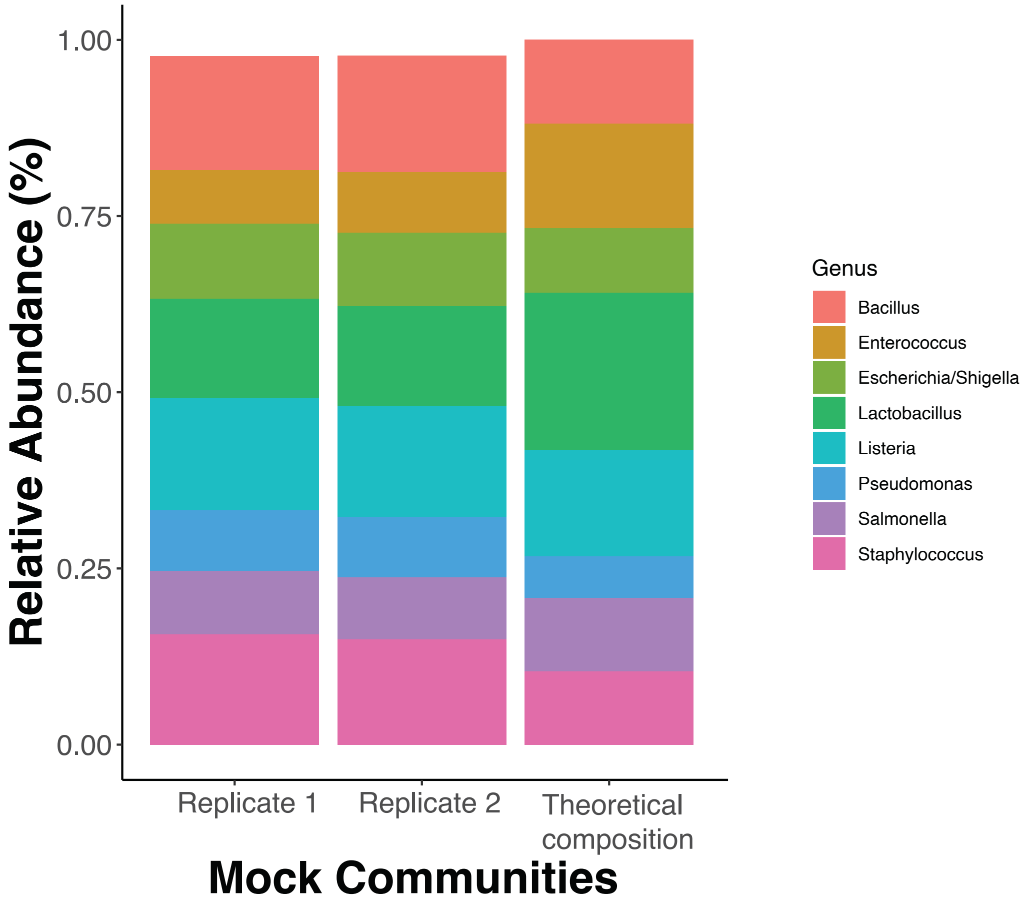

Figure S3. Validation of microbial community transcriptomic workflow using a commercially available ZymoBIOMICS Microbial Community Standard. The mock community consisted of intact cells from eight bacterial genera with a known composition. RNA was extracted from two biological replicates of the mock community (Replicate 1 and Replicate 2), followed by rRNA depletion, library preparation, and sequencing. The resulting reads were processed through the same bioinformatic pipeline used for oyster calcifying fluid samples, including transcriptome assembly and taxonomic annotation. Shown here is the relative abundance of annotated genera in the two replicates compared to the theoretical composition provided by the manufacturer. The close match across replicates and to the expected community supports the accuracy and consistency of the transcriptomic and annotation workflows.

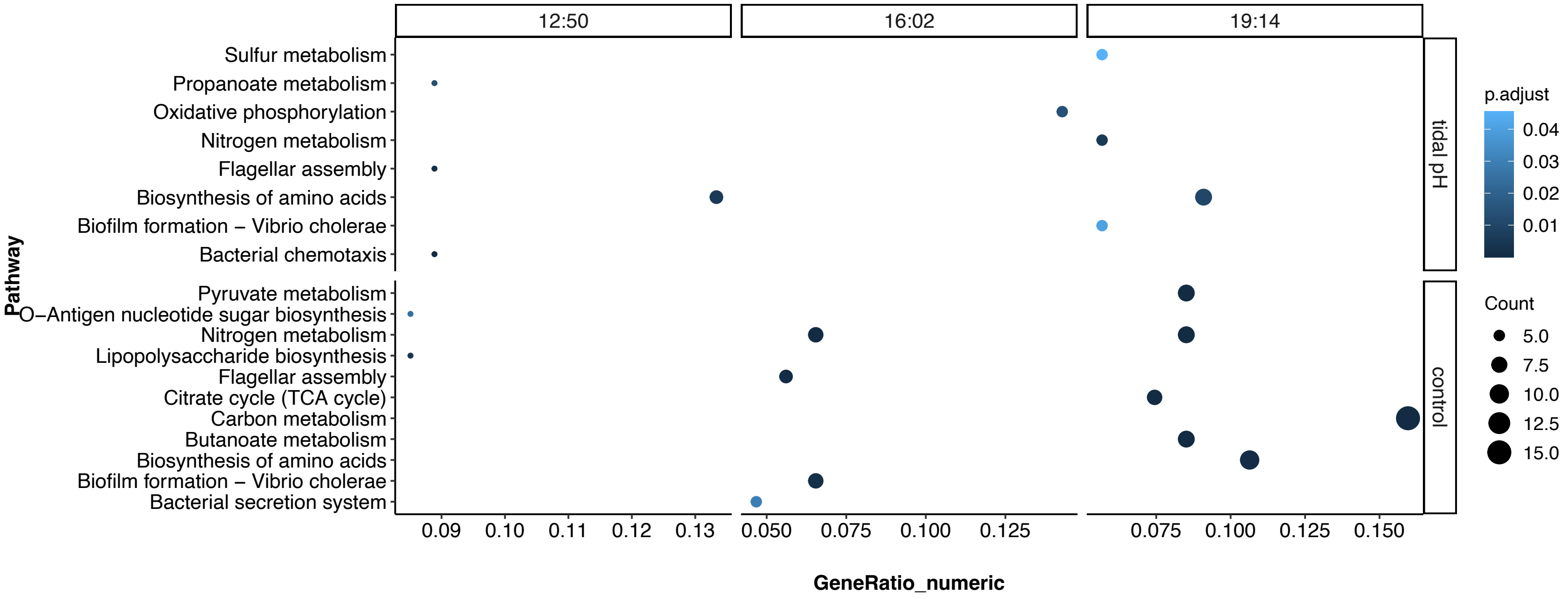

Figure S4. KEGG pathway enrichment analysis of differentially expressed microbial transcripts across treatments and tidal timepoints. Enrichment was performed separately for tidal pH and control conditions at 12:50 (low tide), 16:02 (rising tide), and 19:14 (high tide). Pathways shown were significantly enriched (adjusted $P < 0.05$) based on KEGG annotations of microbial transcripts recovered from oyster calcifying fluid. Dot size indicates the number of differentially expressed transcripts mapped to each pathway, while dot color represents adjusted p-value, with darker shades indicating higher significance. Distinct pathway enrichments were observed between treatments and timepoints, highlighting dynamic microbial metabolic responses to tidal pH fluctuations and potential contributions to host calcifying fluid chemistry.

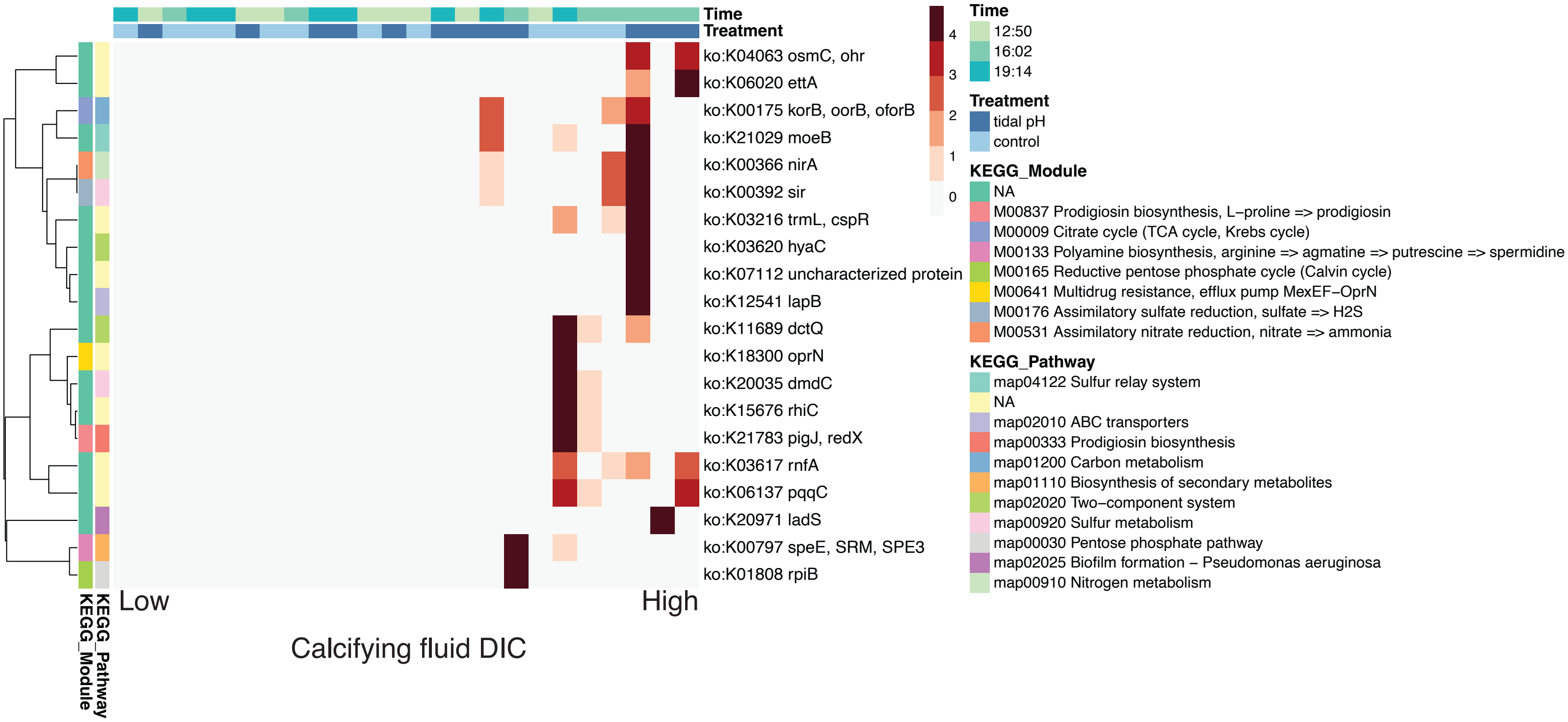

Figure S5. Random Forest regression model identifying microbial transcripts most strongly associated with calcifying fluid DIC. The model was trained using microbial gene expression data from calcifying fluid samples across three tidal timepoints (12:50, 16:02, and 19:14) and both treatment conditions. The heatmap shows expression values of the top microbial transcripts significantly correlated with DIC, with samples ordered along the x-axis by increasing DIC. Rows are annotated with corresponding KEGG module and pathway classifications. Color intensity represents normalized transcript abundance (z-score). Transcripts involved in nitrogen metabolism, sulfur reduction, and carbon cycling were among the most predictive of internal carbonate chemistry, suggesting microbial metabolic activity may contribute to host buffering capacity.

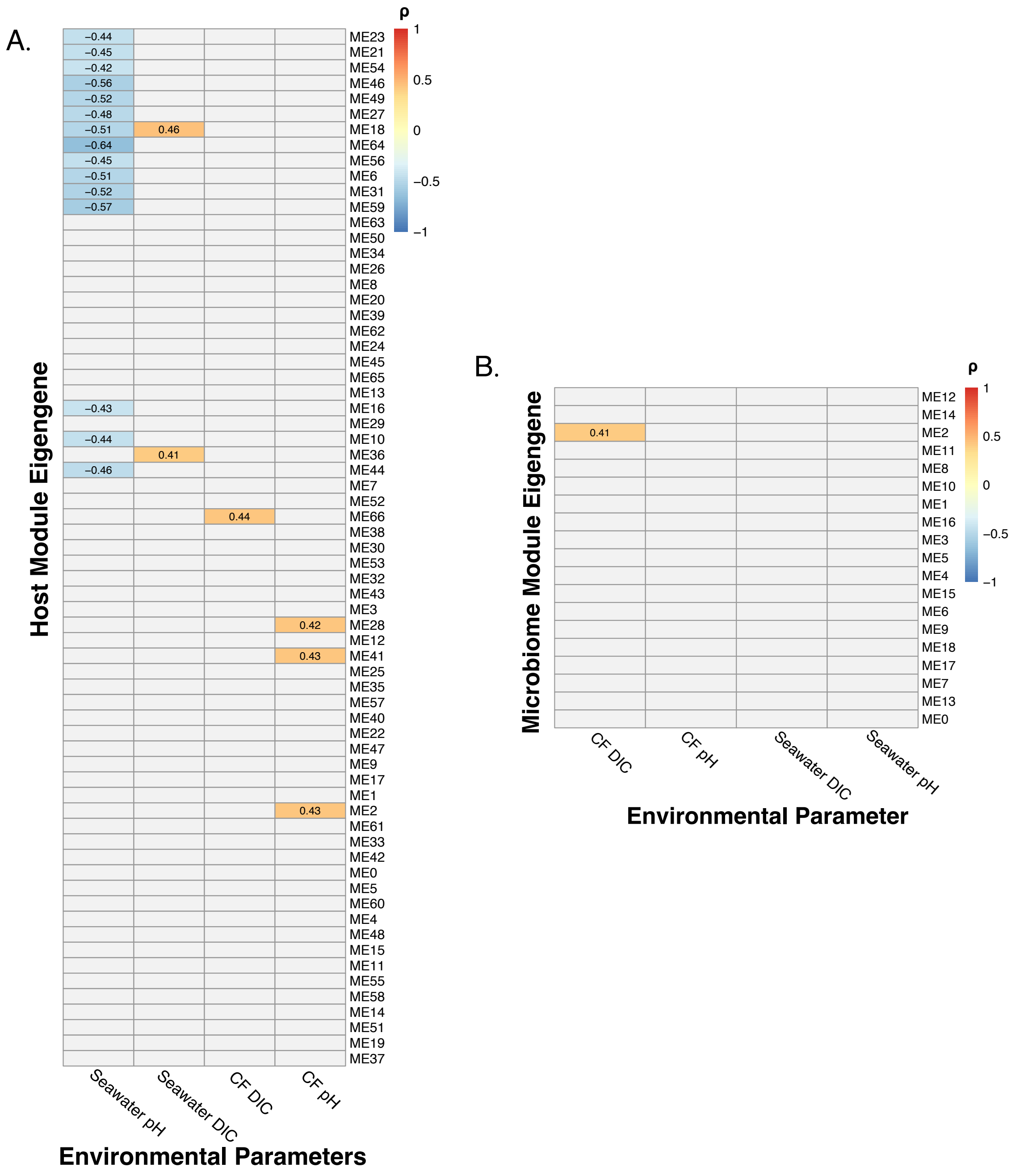

Figure S6. Correlations between host and microbiome module eigengenes and environmental parameters. (**A**) Correlation heatmap showing Pearson correlation coefficients (ρ) between host module eigengenes (rows) and environmental variables (seawater and calcifying-fluid pH and DIC). (**B**) Correlation heatmap showing ρ values for microbiome module eigengenes and the same environmental parameters. Blue indicates negative correlations and orange indicates positive correlations; only significant correlations ($P < 0.05$) are shown. Values within cells represent the correlation coefficient.

SI References

1. Z. Li, M. Yang, C. Zhou, *et al.*, Deciphering the molecular toolkit: Regulatory elements governing shell biomineralization in marine molluscs. *Integr. Zool.* 20, 448–464 (2025).
2. Y. Zhang, Z. Liu, X. Song, S. Huang, L. Wang, L. Song, The inhibition of ocean acidification on the formation of oyster calcified shell by regulating the expression of *Cgchs1* and *Cgchit4*. *Front. Physiol.* 10, 1034 (2019).
3. V. Schönitzer, I. M. Weiss, The structure of mollusc larval shells formed in the presence of the chitin synthase inhibitor nikkomycin Z. *BMC Struct. Biol.* 7, 71 (2007).
4. Z. Liao, L.-F. Bao, M.-H. Fan, *et al.*, In-depth proteomic analysis of nacre, prism, and myostracum of *Mytilus* shell. *J. Proteomics* 122, 26–40 (2015).
5. Y. Zhou, Y. Yan, D. Yang, *et al.*, Cloning, characterization, and functional analysis of chitinase-like protein 1 in the shell of *Pinctada fucata*. *Acta Biochim. Biophys. Sin.* 52, 954–966 (2020).
6. M. Yano, K. Nagai, K. Morimoto, H. Miyamoto, Shematrin: A family of glycine-rich structural proteins in the shell of the pearl oyster *Pinctada fucata*. *Comp. Biochem. Physiol. B* 144, 254–262 (2006).
7. Y. Lin, G. Jia, G. Xu, *et al.*, Cloning and characterization of the shell matrix protein shematrin in scallop *Chlamys farreri*. *Acta Biochim. Biophys. Sin.* 46, 709–719 (2014).
8. C. Zhang, L. Xie, J. Huang, *et al.*, A novel matrix protein family participating in the prismatic layer framework formation of pearl oyster *Pinctada fucata*. *Biochem. Biophys. Res. Commun.* 344, 735–740 (2006).
9. J. Liang, G. Xu, J. Xie, *et al.*, Dual roles of the lysine-rich matrix protein (KRMP)-3 in shell formation of pearl oyster *Pinctada fucata*. *PLoS One* 10, e0131868 (2015).
10. Y. Kong, G. Jing, Z. Yan, *et al.*, Cloning and characterization of Prisilkin-39, a novel matrix protein serving a dual role in prismatic layer formation. *J. Biol. Chem.* 284, 10841–10854 (2009).
11. M. Suzuki, E. Murayama, H. Inoue, *et al.*, Characterization of Prismalin-14, a novel matrix protein from the prismatic layer of *Pinctada fucata*. *Biochem. J.* 382, 205–213 (2004).
12. T. Takeuchi, R. Koyanagi, F. Gyoja, *et al.*, Bivalve-specific gene expansion in the pearl oyster genome: Implications of adaptation to a sessile lifestyle. *Zool. Lett.* 2, 3 (2016).
13. B. Xie, Q. He, R. Hao, *et al.*, Molecular and functional analysis of PmC1qDC in nacre formation of *Pinctada fucata martensii*. *Fish Shellfish Immunol.* 106, 621–627 (2020).
14. X. Xiong, C. Li, Z. Zheng, X. Du, Novel globular C1q domain-containing protein participates in shell formation and immune responses. *Sci. Rep.* 11, 1105 (2021).
15. J. Arivalagan, T. Yarra, B. Marie, *et al.*, Insights from the shell proteome: Biomineralization to adaptation. *Mol. Biol. Evol.* 34, 66–77 (2017).
16. A. Carini, T. Koudelka, A. Tholey, *et al.*, Proteomic investigation of the blue mussel larval shell organic matrix. *J. Struct. Biol.* 208, 107385 (2019).

17. Z. Liao, L.-F. Bao, M.-H. Fan, *et al.*, In-depth proteomic analysis of nacre, prism, and myostracum of *Mytilus* shell. *J. Proteomics* 122, 26–40 (2015).
18. B. Marie, J. Arivalagan, L. Matheron, *et al.*, Deep conservation of bivalve nacre proteins highlighted by shell matrix proteomics. *J. R. Soc. Interface* 14, 20160846 (2017).
19. J. K. Sillanpää, K. Ramesh, F. Melzner, *et al.*, Calcium transfer across the outer mantle epithelium in the Pacific oyster. *Proc. R. Soc. B* 285, 20181676 (2018).
20. K. Ramesh, T. Yarra, M. S. Clark, *et al.*, Expression of calcification-related ion transporters during blue mussel larval development. *Ecol. Evol.* 9, 7157–7172 (2019).
21. W. Fan, C. Li, L. Xie, R. Zhang, Cloning and characterization of calcium channel β-subunit from pearl oyster. *J. Biosci. Bioeng.* 104, 47–54 (2007).
22. W. Fan, C. Li, S. Li, *et al.*, Cloning and expression of sarco/endoplasmic reticulum Ca^{2+}-ATPase isoforms from pearl oyster. *Acta Biochim. Biophys. Sin.* 39, 722–730 (2007).
23. T. Yarra, M. Blaxter, M. S. Clark, A bivalve biomineralization toolbox. *Mol. Biol. Evol.* 38, 4043–4055 (2021).
24. T. Yarra, K. Ramesh, M. Blaxter, *et al.*, Transcriptomic analysis of shell repair and biomineralization in the blue mussel. *BMC Genomics* 22, 437 (2021).
25. A. R. Taylor, A. Chrachri, G. Wheeler, *et al.*, A voltage-gated H^+ channel underlying pH homeostasis in calcifying cells. *PLoS Biol.* 9, e1001085 (2011).
26. L. Zhao, B. R. Schoene, R. Mertz-Kraus, F. Yang, Insights from sodium into bivalve shell formation. *J. Exp. Mar. Biol. Ecol.* 486, 148–154 (2017).
27. H. Miyamoto, T. Miyashita, M. Okushima, *et al.*, A carbonic anhydrase from the nacreous layer in oyster pearls. *Proc. Natl. Acad. Sci. U.S.A.* 93, 9657–9660 (1996).
28. H. Miyamoto, F. Miyoshi, J. Kohno, The carbonic anhydrase domain protein nacrein acts as a negative regulator in calcification. *Zool. Sci.* 22, 311–315 (2005).
29. I. Sarashina, K. Endo, The complete primary structure of molluscan shell protein 1 (MSP-1). *Mar. Biotechnol.* 3, 362–369 (2001).
30. D. Tsukamoto, I. Sarashina, K. Endo, Structure and expression of an unusually acidic matrix protein of pearl oyster shells. *Biochem. Biophys. Res. Commun.* 320, 1175–1180 (2004).
31. Y. Yan, D. Yang, X. Yang, *et al.*, A novel matrix protein, PfY2, functions as a crucial macromolecule during shell formation. *Sci. Rep.* 7, 6021 (2017).
32. L. Yi, B. Zou, L. Xie, R. Zhang, A novel bifunctional protein PNU7 in $CaCO_3$ polymorph formation. *Int. J. Biol. Macromol.* 222, 2796–2807 (2022).
33. M. Suzuki, K. Saruwatari, T. Kogure, *et al.*, An acidic matrix protein, Pif, is a key macromolecule for nacre formation. *Science* 325, 1388–1390 (2009).
34. S.-Y. Bahn, B.-H. Jo, Y.-S. Choi, H.-J. Cha, Control of nacre biomineralization by Pif80 in pearl oyster. *Sci. Adv.* 3, e1700765 (2017).
35. Y. Ji, X. Yang, D. Yang, R. Zhang, PU14, a novel matrix protein, participates in shell formation of *Pinctada fucata*. *Mar. Biotechnol.* 23, 189–200 (2021).

36. D. Yang, Y. Yan, X. Yang, *et al.*, A basic protein, N25, modifies calcium carbonate morphology and shell biomineralization. *J. Biol. Chem.* 294, 8371–8383 (2019).
37. A. Miglioli, R. Dumollard, T. Balbi, *et al.*, Characterization of the main steps in first shell formation in *Mytilus galloprovincialis*. *Proc. R. Soc. B* 286, 20192043 (2019).
38. N. Le Roy, D. J. Jackson, B. Marie, P. Ramos-Silva, F. Marin, The evolution of metazoan α-carbonic anhydrases and their roles in biomineralization. *Front. Zool.* 11, 75 (2014).
